# Reversing Pathology in an Aggravated Fabry Mouse Model Using Low-Dose Engineered Human Alpha-Galactosidase A AAV Gene Therapy

**DOI:** 10.3390/biomedicines13030577

**Published:** 2025-02-25

**Authors:** Wanida Ruangsiriluk, Mugdha Deshpande, Natalia Boukharov, Girija Rajarshi, Shreya Mukherji, Shipeng Yuan, Jennifer Wiseman, Nancy Chen, Eric Park, Hyelim Cho, Rizwana Islam

**Affiliations:** Takeda Pharmaceuticals, Cambridge, MA 02142, USA

**Keywords:** Fabry disease, lysosomal storage disease, adeno-associated virus (AAV), rAAV9, AAV gene therapy, alpha-Galactosidase A (*GLA*), G3Stg/*Gla*KO mouse model, engineered enzyme

## Abstract

**Background/Objectives**: Fabry disease is an X-linked disorder caused by lysosomal accumulation of glycosphingolipids due to the deficiency of α-Galactosidase (α-GAL), which leads to pathology in multiple organ systems. The standard of care is enzyme replacement therapy (ERT) with recombinant native α-GAL protein. Shortcomings of the native α-GAL include low stability, a short circulating half-life, and inadequate uptake by affected tissues that limits the efficacy of ERT and could potentially reduce AAV gene therapy (GT) benefits. Cross-correction by secreted α-GAL is essential for liver-targeted as well as ubiquitous AAV GT due to poor transduction and/or short half-life of some of the significantly affected cell types. **Methods**: To overcome potential limitations of AAV GT delivering native α-GAL, we used an engineered *GLA* transgene product to improve enzyme stability and reduce predicted immunogenicity. **Results**: The stabilized α-GAL variant, Eng-C, had an extended circulatory half-life, allowing for enhanced distribution and efficient uptake by target organs. AAV gene therapy with Eng-C demonstrated significantly greater substrate reduction in the severe Fabry G3Stg/*Gla*KO mouse model across all affected tissues. Efficacy of the Eng-C AVV GT was equal to or greater than the efficacy of the higher doses of the AAV GT with native α-GAL. Furthermore, this study is the first to demonstrate that the pre-existing pathology in some tissues in G3Stg/*Gla*KO mice can be reversed with efficient treatment. **Conclusions**: Our findings demonstrate that an AAV-based gene therapy expressing an engineered α-GAL with improved stability and lower immunogenicity could be effective at lower doses than other AAV GTs, potentially offering lower safety risks typically associated with high AAV vector doses.

## 1. Introduction

Fabry disease is a rare genetic X-linked lysosomal storage disorder caused by mutations in the α-galactosidase A gene (*GLA*) that encodes the ubiquitously expressed α-galactosidase A (α-GAL A) enzyme. The functional deficiency of α-GAL A results in progressive lysosomal accumulation of globotriaosylceramide (Gb3) and its deacylated derivative, globotriaosylsphingosine (lyso-Gb3), leading to multi-organ damage. Clinical manifestations of the disease vary widely due to the broad range of tissues and cell types affected. Early symptoms often include peripheral neuropathy, characterized by excruciating burning pain and episodic crises. Chronic gastrointestinal (GI) disturbances such as diarrhea, constipation, and bloating are also prevalent, significantly impairing quality of life. Renal involvement appears early in life for many male and female patients, and, in some patients, it can progress to end-stage renal disease (ESRD) even with treatment. Cardiac complications, including left ventricular hypertrophy (LVH), fibrosis, and abnormal electrocardiograms, can lead to heart failure or myocardial infarctions. Additionally, Fabry patients frequently experience transient ischemic attacks and strokes at a relatively young age [1].

Current treatment options for Fabry disease include enzyme replacement therapy (ERT) with recombinant human α-GAL A (rh α-GAL A) enzyme preparations and pharmacological chaperone therapy (PCT) for amenable mutations [2]. While these therapies can slow disease progression, they do not address all pathological manifestations. Many patients continue to experience significant symptoms. Moreover, patients often develop anti-drug antibodies, reducing therapeutic efficacy. In addition, the requirement of lifelong biweekly or even weekly ERT infusions is a significant burden for patients, caregivers, and healthcare systems [3,4,5,6]. The limited clinical impact of ERT is partly attributed to poor enzyme stability in neutral pH environments, limited lysosomal half-life, and insufficient access to critical cells in affected organs such as the cardiomyocytes, renal distal tubular cells, and peripheral neurons. Consequently, in many patients, renal function declines, cardiovascular pathology progresses, and neuropathic pain and GI symptoms persist despite treatment. In particular, cardiovascular pathology remains a high unmet clinical need and is the most common cause of mortality, accounting for 75% of all premature deaths in Fabry disease [3,4,5,6].

Over the past decade, more novel therapeutic approaches have sought to overcome the biophysical liabilities inherent with the first generation of systemically administered rh α-GAL A, Fabrazyme^®^ and Replagal^®^, which are produced from mammalian cell lines. The most recently approved ERT for Fabry disease, pegunigalsidase alpha (Elfabrio^®^), was produced from a plant cell line, covalently cross-linked and pegylated. These modifications significantly improved stability and increased the half-life in serum and tissues, but the reported efficacy in Gb3 reduction in animal models and kidney function in patients was comparable to Fabrazyme [7,8,9]. Lack of improved in vivo pharmacology may be attributed to pegylation impacting the degradation of the natural Gb3 glycosphingolipid substrate, in contrast to small synthetic fluorescent or colorimetric reporter substrates typically utilized for quantitative enzyme activity analysis [8]. Additional alternative therapeutic approaches such as combination therapies of recombinant enzymes with a stabilizing small molecule chaperone have been evaluated preclinically and clinically [10,11,12], but unlike for another lysosomal disorder, Pompe disease [13,14], this combination approach has not advanced as a therapeutic option for Fabry patients.

Recently, adeno-associated gene therapies (AAV GTs) for Fabry disease have shown promise in providing a more long-lasting treatment option compared to current therapies. GT candidates such as ST-920 (Sangamo, Brisbane, CA, USA) and 4D-310 (4D MT, Emeryville, CA, USA) have reported promising preclinical and early clinical successes in generating durable supraphysiological serum enzyme levels after a single dose, reducing substrate, stabilizing renal function, and improving cardiovascular function. However, the 4D-310 trial experienced a setback due to adverse events, as three of six patients developed atypical hemolytic uremic syndrome (aHUS) and slight increases in serum substrate levels after ERT discontinuation was observed in a few patients in the ST-920 trial [15,16]. Therefore, questions about the safety and long-term efficacy of AAV GTs remain, and further studies are warranted to find the most effective treatment for patients.

Last year we reported the development of an rAAV9 based GT for Fabry disease, rAAV9-h*GLA*, that expresses native α-GAL utilizing a ubiquitous promoter [17]. This GT was able to prevent structural and functional organ pathology in the aggravated G3Stg/*Gla*KO Fabry mouse model treated with the relatively low dose of 6.25 × 10^12^ vg/kg. The goal of the present study was to develop a next generation low-dose gene therapy that can deliver a stabilized *GLA* variant to affected tissues for both intrinsic expression as well as liver-based production and secretion. Secretion and uptake of enzymes is necessary even in the case of ubiquitously targeted GT for cross-correction of tissues and cell types not transducible with AAV and/or likely to rapidly lose episomal *GLA* transgene due to fast cellular turnover. We selected an engineered α-GAL variant, Eng-C, as described in Hallows et al., 2023 [18], with preserved enzymatic activity but significantly improved stability under neutral as well as low lysosomal pH, resistance to proteolytic cleavage, and reduced predicted immunogenicity. The improved GT candidate, rAAV9-Eng-C, was constructed using the same approach as previously reported [19], and efficacy was evaluated in the same mouse model as rAAV9-h*GLA* in the earlier study [17] and compared with rAAV9-h*GLA*. Here, we show that rAAV9-Eng-C is able to halt disease progression at a 25-fold lower dose than rAAV9-h*GLA*. In tissues where pathology was detected at baseline, this novel gene therapy is also able to fully or partially reverse pathological changes and normalize organ function. These results suggest that rAAV9-Eng-C represents a significant step forward in addressing Fabry disease pathology while reducing the risks associated with high doses often required for AAV-based gene therapies.

## 2. Materials and Methods

### 2.1. Transient Transfection of Plasmids in Huh7 and Hepa1-6

The human hepatocarcinoma cell line (Huh7) was obtained from the Japanese Collection of Research Bioresources Cell Bank, and the mouse hepatocarcinoma cell line (Hepa1-6) was obtained from American Type Culture Collection (ATCC). The cells were maintained at 70–80% confluence in growth media consisting of high-glucose DMEM, 10% fetal bovine serum (FBS), 2 mM L-glutamine, 10 mM 2-[4-(2-hydroxyethyl)piperazin-1-yl] ethanesulfonic acid or 4-(2-Hydroxyethyl)piperazine-1-ethanesulfonic acid (HEPES), and 1% penicillin-streptomycin (Thermo Fisher Scientific, Waltham, MA, USA) at 37 °C, 5% CO_2_. Cells were seeded in a 12-well plate overnight at a density of 125,000 cells per well and transfected the following day, using Lipofectamine 3000 (Thermo Fisher Scientific) complexed with plasmid DNA at a ratio of 1 µg of DNA to 1.5 µL of lipid. Next day, the media were refreshed, and incubation continued for another day. Cell culture media were collected, and transfected cells were washed and lysed before being subjected to the alpha galactosidase activity assay and Bicinchoninic Acid (BCA) protein quantification assay for normalization of cell lysate activity.

### 2.2. Recombinant AAV Vector Production

The vector genome for rAAV9-Eng-C contains a 5′ inverted terminal repeat (ITR), a strong ubiquitous promoter comprising CMV enhancer, CBA (Chicken beta-actin) promoter, and a hybrid rabbit beta globin intron, codon-optimized, modified human α-galactosidase gene (h*GLA*) gene sequence encoding an engineered variant of α-GAL protein, a woodchuck hepatitis virus post-transcriptional regulatory element (WPRE), a bovine growth hormone polyadenylation sequence (bGHpA), and a 3′ ITR. The coding sequence for this transgene was designed using a codon optimization algorithm to improve its expression in human heart, kidney, and liver tissues, while reducing immuno-stimulatory CpG motifs and additional modifications to improve protein expression (e.g., removing repeat and inverted repeat sequences and potential splicing sites) to suit gene therapy application. The null vector genome comprises a non-coding DNA sequence flanked by ITR sequences. All vector genomes contained a short barcode sequence designed for detection of vector genomes by qPCR/ddPCR for the purpose of titering and biodistribution assays. ITR plasmids containing transgene expression cassettes were produced by standard restriction cloning techniques.

The rAAV vectors were produced using standard triple-transfection methods [19]. In brief, HEK293 cells were co-transfected with three plasmids: the ITR plasmid containing the transgene expression cassette, a RepCap expression cassette, and an adenoviral helper gene expression cassette. After 72–96 h, the cells were chemically lysed, and the cell pellet and medium were collected. The cell lysate was clarified and treated with benzonase. The clarified lysate was loaded to a POROS CaptureSelect AAVX column (Thermo Fisher Scientific) connected to an AKTA purification system (Cytiva, Marlborough, MA, USA), and AAV vector-containing fractions of the eluate were collected according to the manufacturer’s recommended conditions. Fractions containing AAV vectors were pooled and subjected to a cesium chloride continuous gradient to enrich for full capsids. Fractions containing mostly full capsids were pooled and subjected to buffer exchange. The AAV vectors were formulated in phosphate buffered saline with 0.001% Pluronic F-68 (Thermo Fisher Scientific) and were subjected to standard characterization, including a quantitative polymerase chain reaction (qPCR) for titering, silver staining for purity, an amebocyte lysate assay (Endosafe^®^, Charles River Laboratories, Wilmington, MA, USA) for endotoxin measurement, and an in vitro transduction assay to determine biological activity. Characterized vectors were aliquoted and frozen at −80 °C.

### 2.3. In Silico Immunogenicity Prediction

Immunogenicity of the variants was characterized in silico using the public Immune Epitope Database (IEDB) and utilizing the stabilization matrix alignment method (SMM-align) [20]. The method allows identification of the MHC II binding motifs and their binding affinity. The immunogenicity score was computed based on the number of epitopes and the associated HLA gene allele frequency. The top 20% binding epitopes with the highest affinity were designated as “Strong”. The top 20% of predicted epitopes can account for 50% of total immunogenicity. The cut-off binding affinity is specific for each allele and was established using an approach similar to one described elsewhere [21].

### 2.4. pH and Human Serum Stability Assay

Purified α-GAL proteins were produced following Hallows et al., 2023 [18]. To assess the relative pH stability of the native and Eng-C α-GAL variant, proteins were diluted into 1× phosphate buffered saline (PBS), pH 6.2 to 50 μg/mL (10×). McIlvaine buffers at the indicated pH were prepared, and 45 μL of McIlvaine buffer at each tested pH level and 5 μL of 10× protein were transferred into the 96-well plate. The sealed plate was incubated at 37 °C for 24 h with agitation at 400 rpm. Residual enzymatic activity was measured at the end of the incubation period using α-GAL enzyme activity described below. Values were normalized to unchallenged conditions.

For α-GAL stability assessment in human serum, 90 μL of pooled human serum (BioIVT, Westbury, NY, USA) was added to enzyme at a concentration of 1 μM in 10 μL PBS (pH 6.2). The 96-well plates were sealed and incubated for the indicated time at 37 °C with agitation at 300 rpm. At the conclusion of each stability challenge, samples were diluted 1:1 into alpha galactosidase activity buffer to assess residual active enzyme. Data were normalized to variant activity in the absence of serum.

### 2.5. In Vitro Evaluation of Cross-Reactivity of the ADA-Positive Fabry Patients’ Serum Samples with Eng-C α-GAL Variant

Fabry patient serum samples were obtained from Sanguine Biosciences (Woburn, MA, USA) with patient consent. Samples were analyzed for anti-drug antibodies (ADAs) against human α-GAL using a Bridge ELISA method. To evaluate the effect of pre-existing ADAs on enzyme activity, 10 µL of 50 ng/mL purified WT α-GAL and Eng-C variant α-GAL proteins (25 ng/mL final concentration) were incubated with 10 µL of 5-fold serial dilutions of patient serum samples (from 1:2 to 1:31,250) for 1 h. Normal human pooled serum (BioIVT, Westbury, NY, USA) was used as a negative control (0% inhibition), and commercial rabbit polyclonal anti-human α-GAL antibody (Novus Biologicals, Centennial, CO, USA) was used as a positive control (100% inhibition). Residual α-GAL activity was measured as described in the α-GAL enzyme activity method section. Percent inhibition was calculated using the activity of the corresponding dilution of healthy control serum as having 100% activity.

### 2.6. α-GAL Enzyme Activity

Alpha galactosidase activity was determined using 4-Methylumbelliferone-α-gal fluorescent (4-MU-α-gal) substrate. Briefly, 2 µL of a biological sample was incubated with 15 μL of 4-MU-α-gal substrate solution (M65400, Research Products International Company, Mt. Prospect, IL, USA) with α-galactosidase B inhibitor (N-acetyl-D-galactosamine A-2795, Sigma-Aldrich Chemicals, St. Louis, MO, USA). The enzymatic reaction was carried out in the citric-phosphate sample reaction buffer, pH 4.6, at 37 °C for 60 min. The enzymatic reaction was stopped by addition of 200 μL glycine carbonate stop solution (133 mM glycine, 83 mM sodium carbonate), pH 10.7 (Sigma-Aldrich Chemicals). The 4-MU product was measured at the excitation wavelength of 360 nm and emission wavelength of 465 nm by a fluorescence plate reader, SpectraMax M5 (Molecular Devices, San Jose, CA, USA). The concentrations of 4-MU in test samples were calculated from the 4-MU calibration curve in the same plate. Serum and tissue α-GAL activity was normalized to the volume of serum or tissue total protein concentration determined by BCA assay and expressed as nmol of 4-MU substrates per hour per mL of serum or mg of total protein.

### 2.7. α-GAL Protein Concentration

α-GAL protein concentration was quantified by a sandwich ELISA using Meso Scale Discovery platform (MSD, Rockville, MD, USA). The antibodies used were goat polyclonal anti α-GAL antibody (AF6146, R&D Systems, Minneapolis, MN, USA) and rabbit polyclonal anti α-GAL antibody (H00002717-D01P, Novus Biologicals, Centennial, CO, USA), or Takeda generated rabbit polyclonal anti-alpha-Galactosidase Eng-C. Briefly, the primary antibody against human α-GAL protein was captured on the plate overnight at 4 °C, then washed and blocked with the blocking buffer. Samples were prepared and incubated for one hour at room temperature. The plate was washed, and detection antibody with Sulfo-tag was incubated for one hour at room temperature before developing the signal with a read buffer. Concentration was extrapolated from a standard curve corresponding to each α-GAL native or variant protein and normalized to volume of serum or tissue total protein concentration determined by BCA assay.

### 2.8. Gb3 and Lyso-Gb3 Quantification

Substrates from serum and tissue samples were analyzed at NovaBioassays LLC (Woburn, MA, USA). Briefly, the method of liquid chromatography mass spectrometry (LC/MS) with Gb3 and lyso-Gb3 internal standards was established and used as a calibrator in the analysis. Samples were extracted first using chloroform:methanol (volume over volume [*v*/*v*] 2:1) and formic acid before running in high performance liquid chromatography (HPLC) and liquid chromatography tandem mass spectrometry (LC/MS/MS) (Applied Biosystem, Foster City, CA, USA, API5000), Turbo Ion Spray Ionization, positive-ion mode. Tissue Gb3 and lyso-Gb3 levels were normalized by total protein concentration determined by the BCA method.

### 2.9. Mouse Biodistribution and Pharmacology Studies and Tissue Processing

The G3Stg/*Gla*KO [22] and *Gla*KO [23] Fabry mouse models colonies were maintained at Taconic Biosciences (Boston, MA, USA). All studies were conducted following the Animal Welfare and Institutional Animal Care and Use Committee Review and were approved by the Takeda Institutional Animal Care and Use Committee (IACUC). Male mice were injected with AAV test articles at the age of 8–12 weeks old and sacrificed at the age indicated in each study. A group of mice sacrificed right before treatment were used as the pre-dose control group. Blood was collected at indicated times via the submandibular route, processed into serum through centrifugation, and evaluated in different assays. Terminal blood was obtained through cardiac puncture and processed into serum. Mice were transcardially perfused with phosphate-buffered saline (PBS, Thermo Fisher Scientific) for 4–5 min or until the liver was cleared of blood prior to tissue collection. Tissues and serum for analytical endpoints were harvested and snapped frozen. For histology, tissues were processed as described in the histology method section.

Tissues collected for total protein concentration, α-GAL activity, and Gb3 and lysoGb3 substrate levels were subjected to homogenization using Precellys24 system (Bertin Instruments, France Rockville, MD, USA) with appropriately sized beads and tubes and volumes of lysis buffer comprised of 10 mM HEPES, 0.5% TritonX-100, 1.5× protease inhibitors (Thermo Fisher Scientific). Supernatants from tissues were collected through centrifugation at 4 °C for 15 min and evaluated in all the assays mentioned above.

### 2.10. NHP PK and Biodistribution Study

The pharmacokinetic profile of α-GAL A in plasma was evaluated in a 3-month study in non-human primates (NHPs). Animals were dosed with rAAV9-Eng-C at 6.25 × 10^12^ vg/kg (Group 2: 2 females, 1 male) and 3 × 10^13^ vg/kg (Group 3: 4 females, 3 males). Two animals were injected with buffer and used as controls. rAAV9-Eng-C intravenous infusion (target 30 min) was conducted via a suitable peripheral vein using an infusion pump connected to a syringe with an infusion line and a temporary in-dwelling catheter. Bioanalytical blood samples were collected on Day 1 (pre-dose), Day 3, Day 7, and then weekly. Samples were allowed to clot at ambient temperature and then centrifuged. The resultant serum was separated, split into the aliquots, and stored at −80 °C before analysis. Serum samples were analyzed for α-Gal transgene product, anti-rAAV9 total anti-drug antibodies (TAb ADAs), transgene product ADAs, and anti-AAV9 neutralizing antibodies (NAbs), using qualified analytical procedures. In-cage health observations were conducted daily. Clinical evaluations were performed once a week.

### 2.11. Detection of Anti-α-GAL Anti-Drug Antibodies (ADAs), Total Anti-Capsid ADAs (TAb) and Neutralizing Anti-Capsid Antibodies (NAbs)

The analytical method to assess α-GAL anti-drug antibodies (ADAs) was based on a homogeneous electrochemiluminescence (ECL) bridging assay, as described earlier [19]. Detection was based on bridging of biotin- and ruthenium-labeled recombinant human α-GAL (Takeda Pharmaceuticals USA, Lexington, MA, USA) by ADAs in test samples.

#### 2.11.1. rAAV9 Capsid ADA Assay

For the rAAV9 capsid ADA assay (a sandwich ECL assay), serum samples were diluted to the minimum required dilution and added to an AAV9-coated plate; after incubation for 1 h, the plate was washed, and the rAAV9 ADA bound to the AAV9 capsid was detected with SULFO-TAG-labeled Protein A/G/L (Meso Scale Diagnostics, Rockville, MD, USA).

#### 2.11.2. rAAV9 Capsid NAb Assay

The rAAV9 capsid NAb assay was a cell-based assay that used a rAAV9 vector coding for luciferase as a reporter. Briefly, serum samples were first incubated with an rAAV9-CMV-*Luc* vector for 1 h, then the mixture was added to wells containing pre-seeded HEK293T cells, obtained from ATCC, pretreated with etoposide. The cells were lysed after 2 days, and the luciferase in the cell lysate was measured. The luciferase signal intensity inversely correlates with the concentration of NAb in the serum sample. The assay threshold was established at a 50% reduction in luciferase signal, and any sample with a signal reduction of more than 50% was determined as NAb positive.

### 2.12. Wild-Type Mouse Dose Range-Finding Safety Study

A preliminary safety assessment was conducted in 7–9-week-old C57BL/6 male mice in a 6-week non-GLP dose range-finding study. Male animals, 6/cohort, were assigned randomly to 4 cohorts: vehicle buffer, 6 × 10^12^ vg/kg, 3 × 10^13^ vg/kg, and 1 × 10^14^ vg/kg. Animals received a single intravenous tail vein injection of rAAV9-Eng-C or vehicle in a dose volume of 10 mL/kg on day 1. Toxicological endpoints included clinical observations, body weights, body weight gains, food consumption, clinical pathology and anatomic pathology. Clinical observations were conducted pre-dose, on study day 1, and daily from days 2 to 43 for all animals to assess for moribundity and mortality. Individual body weights were collected pre-dose, on days 1, 2, 3, and 4, then weekly thereafter until terminal body weight at necropsy. Food consumption was measured for a quantitative assessment on a weekly basis since the animals were group-housed. Animals were euthanized via carbon dioxide asphyxiation on day 43 post-dose rAAV9-Eng-C or vehicle, and tissues were evaluated macroscopically. Whole blood was collected via cardiac puncture for hematology and serum chemistry sample analysis. For serum chemistry, blood was collected into tubes containing no anticoagulant, allowed to clot, then centrifuged to obtain serum and frozen until use. For hematology, blood was collected into tubes containing K_2_EDTA and refrigerated until use. For hematology and serum chemistry, all samples were sent to Idexx BioAnalytics (North Grafton, MA, USA) for analysis. Whole blood for toxicokinetics was collected pre-dose and at terminal necropsy (Day 43) from all animals via the facial or tail vein into tubes containing K_2_EDTA. Samples for toxicokinetics were analyzed for α-GAL activity using the method described above.

### 2.13. Mouse Sensitivity to Pain Response (Hot Plate Latency)

Sensitivity to pain was measured in each mouse over the duration of the study by placing the mice on a heated surface (hot plate) following a well-established method. Briefly, mice were tested pre-dose (week 10) and subsequently at 2-, 4-, 8-, 12-, and 16-weeks post-dose. Mice were placed on a hot plate (Columbus Instruments, Columbus, OH, USA) preheated to 55 °C inside an open-ended cylindrical plexiglass tube with a diameter of 30 cm to prevent escape. Sensitivity was measured as the time from placement on the heated surface to the first pain response (paw-lick, jump). Mice with no pain response were removed automatically after 1 min to prevent tissue damage. Scoring of the first pain response was blinded to the treatment group to prevent bias.

### 2.14. Genomic DNA Isolation

Approximately 50 mg of tissues aliquoted into Precellys CK14 lysing tubes (Bertin Instruments) were subjected to DNA extraction. The MagMAX™ DNA Multi-sample Ultra 2.0 kit combined with an automated Kingfisher flex magnetic particle processor (Thermo Fisher Scientific) was used for genomic DNA extraction with modification. Briefly, 1 mL of tissue lysis buffer containing β-mercaptoethanol was added to the samples and homogenized in a Precellys bead beater (Bertin Instruments). The homogenization cycle was repeated twice to ensure complete homogenization of the tissue. Tissue lysate was transferred to a 96-well (deep well) plate, then enhancer solution and proteinase K were added and incubated at 65 °C overnight. Next day, RNase of A was added and mixed for 5 min at 900 rpm at room temperature to keep RNA contamination negligible. The bead mixture was added onto each well, and lysates were immediately processed for DNA extraction using the automated Kingfisher flex magnetic particle processor program (MagMAX_Ultra 2_Tissue_V). Genomic DNA concentrations and purity of extracted DNA were monitored by a Lunatic UV/Vis absorbance spectrometer (Unchained Labs, Brighton, MA, USA).

### 2.15. Vector Genome Quantification

The genomic DNA was added to the ddPCR reactive mix containing the respective primers and probes for an un-transcribed region between ITRs and mouse α-Actin as a reference. Droplets were generated using the automated droplet generator (Bio-Rad Laboratories, Hercules, CA, USA). Following PCR amplification of the target sequence by end-point PCR in each droplet, the positive droplets were then quantified using the QX200 droplet reader (Bio-Rad Laboratories). After the assay completion, the threshold was manually set up, and the viral genome copy number was calculated.

### 2.16. mRNA Quantification

Total RNA was isolated from the tissue using a similar method as genomic DNA isolation, using a MagMAX™ total RNA isolation kit (Thermo Fisher Scientific), following the manufacturer’s protocol.

For the *GLA* mRNA copy number, total RNA was subjected to ddPCR. Briefly, an equal amount of total RNA input was added to a reaction containing Supermix, reverse transcriptase, DTT, primers, and a probe against the WPRE transcribed region before loading into GCR96 cartridges and run in the QXONE system (Bio-Rad Laboratories). After the assay was completed, a threshold was manually established, and the mRNA copy number was calculated and normalized by total RNA input per well.

### 2.17. Measurement of Blood Urea Nitrogen (BUN), Urine Albumin, and Creatinine Levels

For measurements of albumin and creatinine levels in urine, and BUN in the blood, samples were analyzed using a COBAS C 311 analyzer (Roche Diagnostics, Indianapolis, IN, USA). Serum BUN (Roche Diagnostics, Catalog No. 04460715 190) was determined by enzymatic reactions with urease dehydrogenase and glutamate dehydrogenase, according to the manufacturer’s protocol. For urine albumin, ALBT2 (Roche Diagnostics, Catalog No. 04469658 190), an immunoturbidimetric assay using an anti-albumin antibody to form antigen/antibody complexes following agglutination was utilized, where turbidimetry was measured. Urine creatinine was analyzed in the CREJ2 (Roche Diagnostics, Catalog No. 04810716 190) assay, which is a kinetic colorimetric assay based on the Jaffé method. Urine albumin concentration was normalized to urine creatinine for final values.

### 2.18. Tissue Sample Collection, Paraffin-Embedded Tissue Block Preparation and Histology

Following perfusion, mouse organs were quickly dissected and rinsed with cold phosphate buffered saline (PBS), then fixed in neutral buffered formalin (NBF) (Thermo Fisher Scientific, Catalog No. SF100-20) with an excess volume of fixative, shaking on an orbital shaker at room temperature for 48 h before being washed and transferred to PBS. The spinal column was collected between the base of the neck and the base of the tail with the spinal cord intact. Mouse foot pads were collected as a whole foot and separated between back footpads. Samples containing bone tissues were subjected to decalcification with RapidCal Immuno Decalcifier (BBC Biochemical, Mt Vernon, WA, USA, Catalog No. 6089) following fixation on an orbital shaker at room temperature for another 24 h. Then, the decalcified samples were rinsed in running RODI (reverse osmosis deionized) water for 1 to 2 h. All fixed tissue samples were processed for paraffin-embedded blocks by a Tissue TEK VIP Tissue processor (SAKURA Finetek USA, Inc., Torrance, CA, USA). Five-micron tissue sections were collected for immunohistochemistry (IHC) assay, performed on an automated Leica Bond system (Leica Biosystems, Deer Park, IL, USA) using a Bond Polymer Refine Detection kit (Leica Biosystems). A brief description of the Leica standard mouse or rabbit antibody protocol is as follows: sections were pre-treated for 20 min using heat-mediated antigen retrieval with bond epitope retrieval solution 1, pH 6.0 (Leica Biosystems, Catalog No. AR9961). Antibodies against the following targets were obtained as indicated: α-GAL (Takeda Pharmaceutical, Cambridge, MA, USA), Collagen I (Boster Bio, Pleasanton, CA, USA, Catalog No. PA2140-2), LAMP1 (Abcam, Waltham, MA, USA, Catalog No. ab24170), PGP9.5 (Abcam, Catalog No. ab8189), beta-catenin (Abcam, Catalog No. ab32572), myelin protein zero (MPZ, Abcam, Catalog No. ab183868). Bond epitope retrieval solution 2 (pH 9.0, Leica Biosystems, Catalog No. AR9640) was incubated with each antibody for 30 min at room temperature and detected using a horseradish peroxidase (HRP) conjugated compact polymer system. 3,3′-diaminobenzidine (DAB) was used as the chromogen, and the sections were then counterstained with hematoxylin. The stained slides were dehydrated and mounted with Cytoseal*60 Mounting Medium (Thermo Fisher Scientific) and coverslips by a Leica CV5030 Fully Automated Glass Coverslipper (Leica Biosystems). Hematoxylin and eosin (H&E) staining was conducted by an Automated Leica ST5020CV5030 Stainer Integrated Workstation (Leica Biosystems). All stained slides were scanned with a Leica AT2 Scanner (Leica Biosystems). Aperio ImageScope and HALO^®^ image system (Indica Labs, Albuquerque, NM, USA) were used for digital images viewing, and all images were viewed, and representative images were selected for figures. The whole slide images were analyzed with HALO^®^ image analysis software version 4.0 (Indica Labs). Data were plotted and analyzed in GraphPad Prism 10.2.1 (GraphPad Software, La Jolla, CA, USA). A positive pixel count algorithm was calibrated for MPZ or LAMP1 positive staining for image analysis. The marker positivity was calculated based on the formula positivity (%) = positive area (pixels)/total analyzed area (pixels) × 100%.

### 2.19. Data Analysis

Data were analyzed using GraphPad Prism 10.2.1 (GraphPad Software). Data are presented as the average ± SEM mean. Appropriate analysis methods were used for each data set. Methods included analysis of variance (ANOVA) followed by a Dunnett’s post-hoc test to determine differences between groups, unpaired two-tailed T-test, multiple unpaired T-test, Michaelis–Menten, Pearson and Spearman correlation, Fishers Least Significant Difference (LSD) test, and a nonlinear fit regression model. Methods for each analysis are specified in the corresponding figure legend.

## 3. Results

### 3.1. In Vitro Characterization of a Novel α-GAL Variant Selected for Gene Therapy Development

#### 3.1.1. Transient Transfection and Enzyme Activity

**Figure 1:**
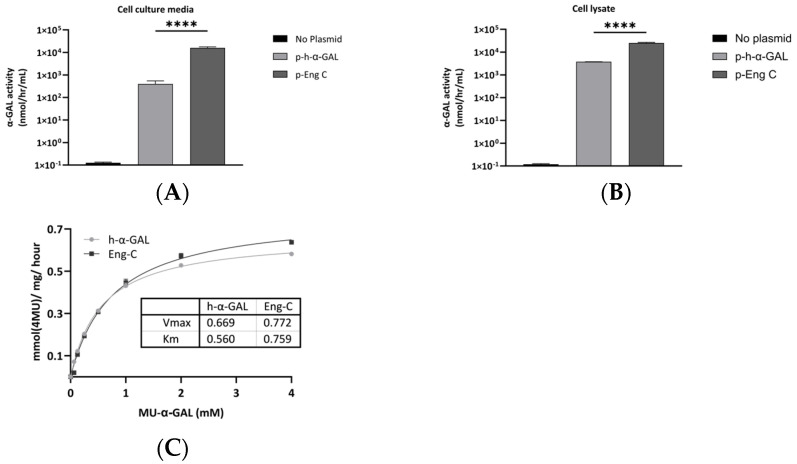
α-GAL expression in transiently transfected Huh7 cells. Huh7 cells were transfected with plasmids carrying either the gene for selected α-GAL engineered variant Eng-C or native α-GAL control. Two days after transfection, cell culture media and cell lysates were tested for α-GAL activity using synthetic 4-MU-α-gal fluorescent substrate. (**A**) Cell culture media. (**B**) Cell homogenate. Activity of Eng-C was compared to that of native α-GAL control using GraphPad Prism 10.2.1 unpaired two-tailed T-test. Both secreted (cell culture media) and cellular (cell homogenate) α-GAL activity of the Eng-C were higher than the native control. **** *p* < 0.0001; *n* = 3. (**C**) Kinetic parameters of the h-α-GAL and Eng-C were compared by measuring enzymatic hydrolysis of the 4MU-α-gal synthetic substrate. Michaelis–Menten analysis was performed using GraphPad Prism 10.2.1.

α-GAL variants were identified by directed evolution and characterized in vitro for serum, pH, and thermal stability by the partner, as described in Hallows et al., 2023 [18]. The Eng-C variant selected for gene therapy development was further characterized. Enzyme expression and secretion was evaluated in Huh7 cells transiently transfected with plasmids containing the coding sequence for either native *GLA* or the Eng-C variant. α-GAL activity was detected in both culture media (Figure 1A) and cell lysate (Figure 1B) and was much higher in samples transfected with Eng-C, consistent with increased stability of the variant. The catalytic activities of the purified Eng-C variant and native α-GAL proteins were similar, indicating that enzyme optimization did not alter Eng-C catalytic properties (Figure 1C). The stability of native *GLA* and the Eng-C variant was assessed by analysis of their residual activity following exposure to human serum or lysosomal-like conditions. Serum stability of Eng-C was significantly higher. After 1 h, the serum incubation activity of Eng-C was reduced by 10%, while native *GLA* lost half of the baseline activity. After 6 h of serum incubation, the activity of native *GLA* was below 5%, while Eng-C still retained 25% of the activity (Figure S1A). The pH of the lysosomal lumen has been reported to range from 4 to 5 [24]. Sphingolipids accumulation leads to an increase of lysosomal pH [25]; therefore, we extended the test range to include pH 6. After 24 h incubation at pH 4, 4.5, and 6, the remaining activity of the Eng-C variant was significantly higher, indicating improved lysosomal stability. At pH 5, the stability of native *GLA* and the Eng-C variant were similar (Figure S1B).

#### 3.1.2. Immunogenicity Risk Assessment

**Figure 2:**
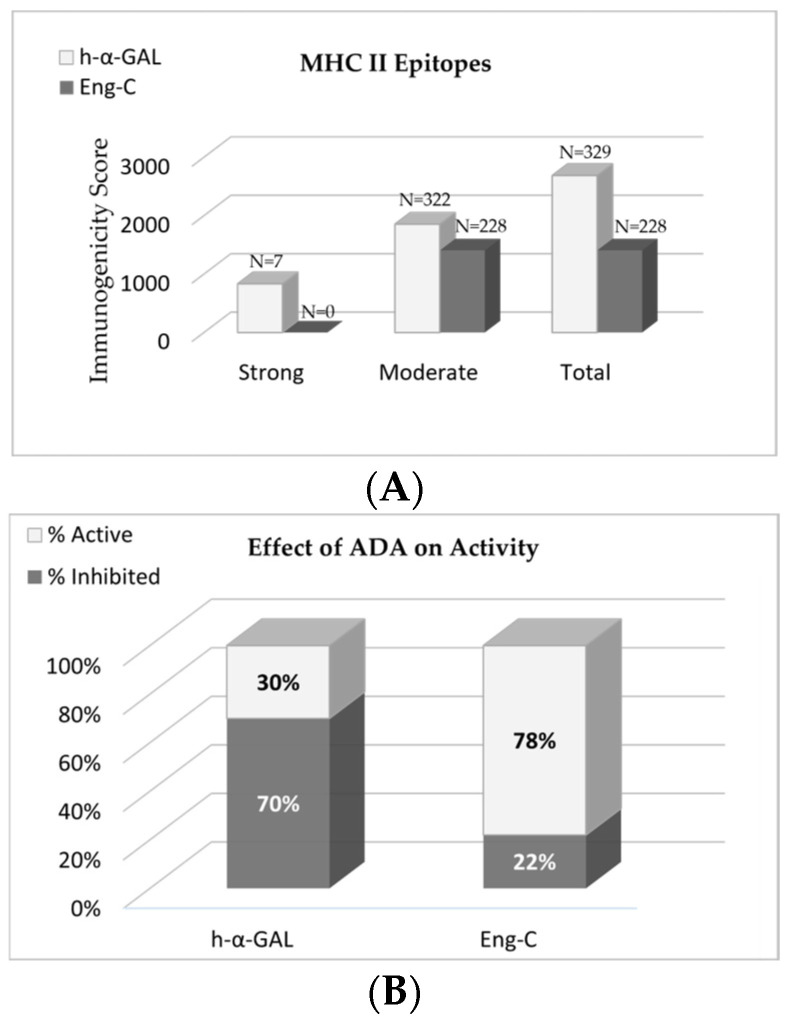
Immunogenicity assessment. (**A**) MHC II epitopes were identified, and the in silico immunogenicity score was calculated using IEDB and SMM-align. The score is based on the number of predicted epitopes within a protein sequence and the frequency of the corresponding HLA allele. The top 20% of binders for each MHC II allele were classified as strong. N = number of epitopes. (**B**) Inhibition of α-GAL activity by ADA in Fabry patient serum was measured at 1:50 dilution. % inhibited and % active were determined using healthy volunteer pooled serum as a 100% activity benchmark.

Immunogenicity risk of the Eng-C variant and native α-GAL was assessed in silico using the public Immune Epitope Database (IEDB) and utilizing the stabilization matrix alignment method (SMM-align) [20]. Amino acid substitutions in the Eng-C variant eliminated all strong immunogenic epitopes found in native α-GAL and reduced 30% of the moderate motifs, significantly lowering the total immunogenicity score (Figure 2A). Cross-reactivity of the Eng-C variant to pre-existing anti-drug antibodies (ADAs) against therapeutic recombinant human native α-GAL was assessed using samples of ERT-treated patients and healthy control serum. Native recombinant α-GAL and Eng-C proteins were pre-incubated with serially diluted serum samples followed by the α-GAL enzymatic activity assay. Activity of both proteins was similar after incubation with either healthy volunteer serum or ADA negative serum sample #64. At a 1:50 dilution, ADA positive sample #65 did not inhibit Eng-C activity, while native α-GAL activity was inhibited by 60%. ADA positive sample #66 did not have any effect on Eng-C activity at 1:250 dilution, while native α-GAL was inhibited by 40% at this dilution (Supplemental Figure S1C). Overall, the average inhibition of the Eng-C activity by pre-existing Fabry patients’ ADAs was significantly lower than the inhibition of activity of the native α-GAL (Figure 2B).

### 3.2. Improved In Vitro Attibutes of the Eng-C Variant Translated into Better In Vivo Efficacy of the AAV-Based Gene Therapy

#### 3.2.1. Administration of rAAV9-Eng-C to G3Stg/*Gla*KO Mice Resulted in Higher Plasma and Tissue α-GAL Activity than Treatment with rAAV9-h*GLA*

Previously, we reported that administration of rAAV9-h*GLA* encoding native human α-GAL increased plasma and tissue α-GAL activity and reduced Gb3 and lyso-Gb3 in serum and tissues of the severe Fabry G3Stg/*Gla*KO mouse model [22]. To determine if the Eng-C variant further enhanced gene therapy efficacy, we constructed rAAV9-Eng-C using the same approach as in the earlier study [17] and administered this construct to G3Stg/*Gla*KO mice at 5 × 10^10^ vg/kg (low) or 2.5 × 10^11^ vg/kg (high) doses using rAAV9-h*GLA* as a comparator at the same dose levels (Study #1). Four weeks after dosing, mice were sacrificed, and serum, liver, kidneys, and heart were collected for analysis. A dose-dependent increase in vector DNA copy number was observed in the liver and heart but was below the assay detection limit in kidneys of most animals in the low-dose group. Dose-dependent mRNA levels were detected in all tissues at both dose levels. As expected at the low doses used in this study, there was significant animal to animal variability in transduction, but, overall, there was no statistically significant difference in vector genome or mRNA levels between rAAV9-Eng-C and rAAV9-h*GLA* treated animals (Supplemental Figure S2A,B). Liver had a 4- to 350-fold higher vector genome level than other tissues. In addition, transcription capacity of the liver is significantly higher than other tissues, which resulted in 500- to 1000-fold higher mRNA levels in the liver than in the heart or kidneys. A good correlation between vector genome copy number and mRNA level was observed in both groups, with R^2^ > 0.9 and *p* < 0.0001. There was no statistical difference in the regression slope, further supporting comparable mRNA production per genome copy number for both GT vectors (Supplemental Figure S2C).

**Figure 3:**
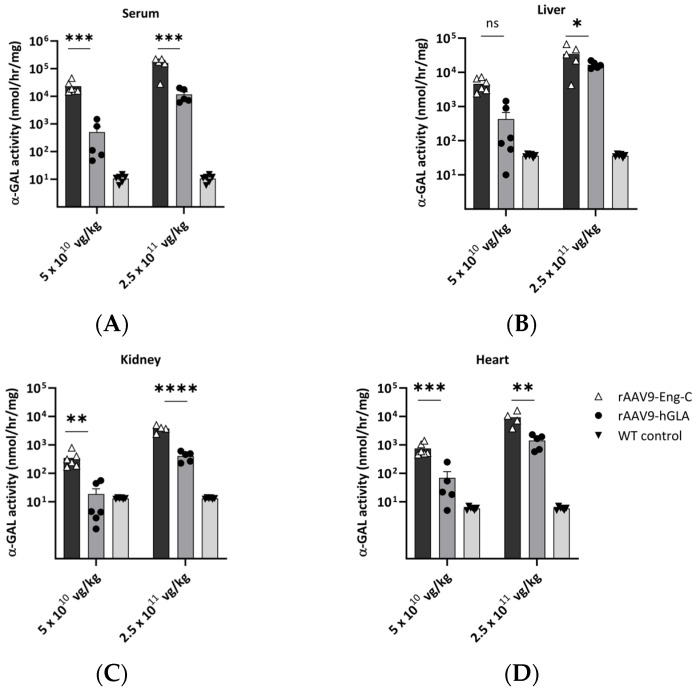
Terminal serum and tissue α-GAL activity. Terminal serum (**A**), liver (**B**), kidney (**C**), and heart (**D**) α-GAL activity was measured using 4-MU-α-gal fluorescent substrate. A dose-dependent increase in activity was observed in all tissue and was significantly higher in animals treated with equivalent doses of rAAV9-Eng-C when compared to rAAV9-h*GLA*. Activity of the rAAV9-Eng-C treated group was compared to rAAV9-h*GLA* using multiple unpaired *t*-tests in GraphPad Prism 10.2.1, ns > 0.05, * *p* < 0.05, ** *p* < 0.01, *** *p* < 0.001, **** *p* < 0.0001; *n* = 5–6.

Consistent with the increased stability of Eng-C observed in vitro and in cell-based assays, higher α-GAL activity was detected in serum and tissues of animals treated with rAAV9-Eng-C (Figure 3A–D). A dose-dependent increase in serum α-GAL activity was observed in treated animals at 1 week after dosing. The level further increased at 2 weeks and was maintained throughout the duration of the study (Supplemental Figure S3).

It has been established that a supraphysiological serum level of α-GAL is required to achieve gene therapy efficacy [26], and the safety of high α-GAL levels are supported by preclinical data from multiple AAV GT therapies and transgenic mouse lines where tissue α-GAL levels >10,000 times endogenous levels did not have any detrimental effect on the animals’ health [27,28]. In our study, treatment with both GT constructs resulted in a 12- to 15,000-fold increase of wild type α-GAL levels in serum and tissues of treated mice (Supplemental Table S1). However, α-GAL activities in animals treated with rAAV9-Eng-C were 6- to 19-fold higher in tissues and 15- to 55-fold higher in serum than in animals treated with rAAV9-h*GLA*, indicating translation of the in vitro stability into improved in vivo PK. The only exception was the liver in the high dose groups, where rAAV9-Eng-C treatment resulted in only a 2-fold increase in α-GAL activity compared to rAAV9-h*GLA*, likely due to saturation of liver lysosomal transport and re-uptake, or proficient secretion of the transgene product Eng-C (Supplemental Table S1). We found a good linear Pearson correlation between serum α-GAL activity with α-GAL activities in the liver, kidney, and heart for both groups. Regression slope coefficients for the serum and kidney and serum and heart α-GAL activities were not different between rAAV9-Eng-C- and AAV9-h*GLA*-treated groups. But the difference in regression slopes for serum and liver α-GAL activity was consistent with saturation of α-GAL lysosomal delivery and/or re-uptake and higher α-GAL fractional secretion in the rAAV9-Eng-C-treated animals (Supplemental Figure S4A–C, Supplemental Table S2). There was also a significant correlation between liver vector genome copy number and serum, kidney, and heart α-GAL activities, with Pearson correlation coefficients between 0.87 and 0.99 for both treatments. At the same time, correlation between heart vector genome (VG) and heart α-GAL activity was low or not significant (Supplemental Table S2). These results indicate that at such low doses, contribution of in situ-produced α-GAL to overall heart exposure is not substantial, and efficacy is mostly achieved through cross-correction by liver-produced enzymes.

Consistent with the higher α-GAL activity achieved at the same transduction level in the rAAV-Eng-C-treated animals, the slope coefficients of the regression model for cardiac and kidney α-GAL activity versus liver VG were significantly greater for the rAAV9-Eng-C group, suggesting that rAAV-Eng-C can attain better tissue α-GAL exposure with lower liver AAV transduction levels, potentially providing better safety of a lower dose treatment (Supplemental Figures S3 and S4D–G).

#### 3.2.2. Treatment with rAAV9-Eng-C Was More Efficient in Clearing Substrate in G3Stg/*Gla*KO Mice than rAAV9-h*GLA*

**Figure 4:**
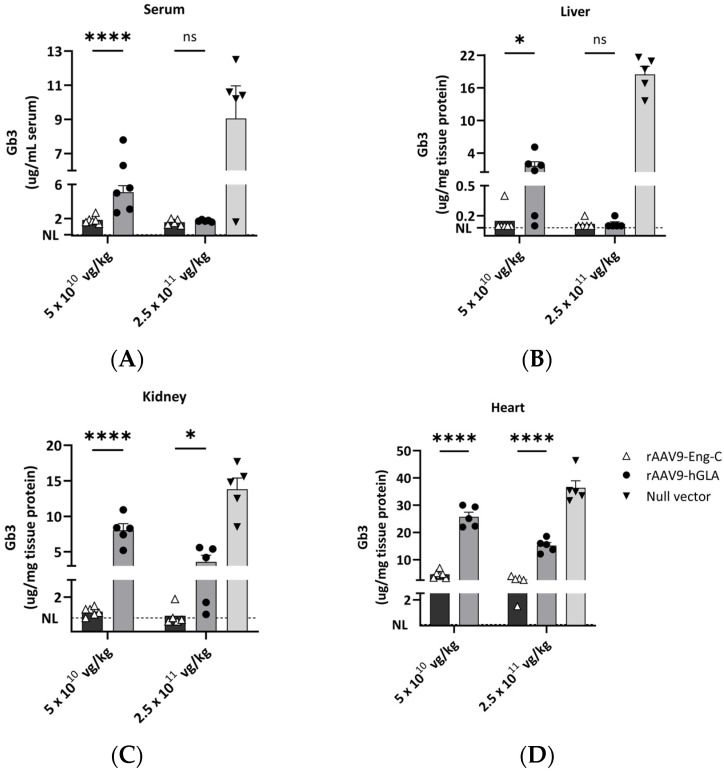
Terminal serum and tissue Gb3 level. Gb3 was measured using LS/MS. Dose-dependent reduction in Gb3 level was observed in terminal serum (**A**) and all tissues, including liver (**B**), kidney (**C**), and heart (**D**). Efficacy was significantly higher in animals treated with the low dose of the rAAV9-Eng-C when compared to the corresponding dose of the rAAV9-h*GLA*. In the high-dose group, rAAV9-Eng-C was more efficient in the heart and kidney. Gb3 in the rAAV9-Eng-C-treated group was compared to rAAV9-h*GLA* using multiple unpaired *t*-tests in GraphPad Prism 10.2.1, ns > 0.05, * *p* < 0.05, **** *p* < 0.0001; *n* = 5–6. NL—normal level.

Next, we sought to determine whether higher α-GAL levels in mice treated with rAAV9-Eng-C translated into increased efficacy. We observed dose-dependent Gb3 clearance, with significantly better reduction in animals treated with rAAV9-Eng-C at both doses. An exception to this were the serum and liver in the high dose groups, where both rAAV9-Eng-C and AAV9-h*GLA* resulted in near complete clearance of Gb3 (Figure 4A–D). While α-GAL activity in the kidney for rAAV9-h*GLA* at the high dose was similar or even higher than the activity conferred by rAAV9-Eng-C at the low dose (Figure 3), the substrate reduction capacity was superior for rAAV9-EngC at the lower dose. Similar findings were observed with the heart data. Results for lyso-Gb3 were consistent with the reductions observed for Gb3 (Supplemental Table S4). These findings demonstrate that the improved stability of Eng-C translated into significant improvement in rAAV9-Eng-C efficacy over AAV9-h*GLA* at equivalent doses.

### 3.3. Treatment with rAAV9-Eng-C Halted Disease Progression and Reversed Pre-Existing Pathology in G3Stg/GlaKO Mice

#### 3.3.1. Systemic Injection of rAAV9-Eng-C Resulted in Dose-Dependent α-GAL A Expression Sustained for 18 Weeks

**Figure 5:**
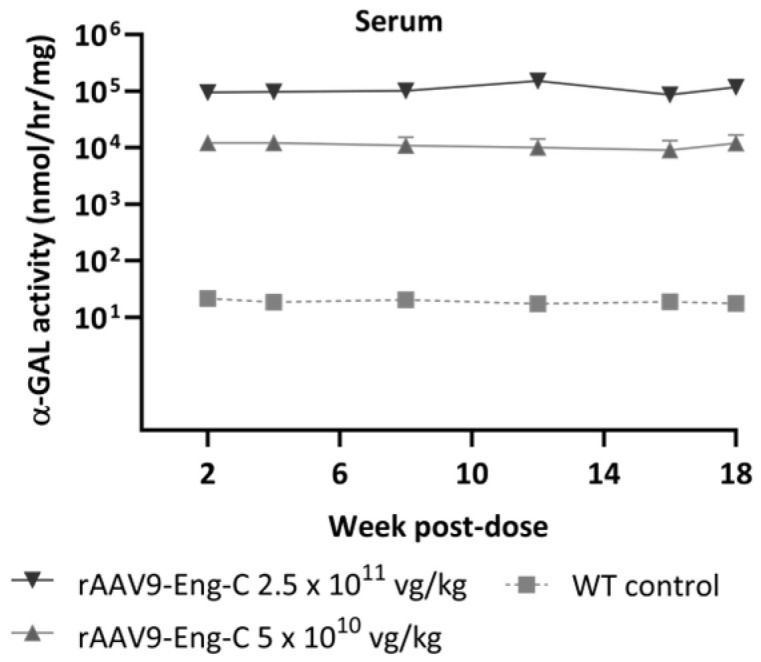
Serum α-GAL activity over time. Serum samples were collected at 2, 4, 8, 12, 16, and 18 weeks after dosing. Activity was measured using 4-MU-α-Gal fluorescent substrate.

Symptomatic G3Stg/*Gla*KO mice accumulate higher substrate levels compared to *Gla*KO mice and develop early Fabry pathology [22]. We performed a detailed characterization of this model [29] to evaluate the progression of different pathological changes and determine the optimal age to test the efficacy of novel therapeutics. We found that by 10 to 12 weeks, G3Stg/*Gla*KO mice develop some Fabry-relevant pathologies. Initiating treatment at this time could allow us to monitor efficacy in halting disease progression, as well as in reversing the pre-existing pathology. To investigate whether treatment with rAAV9-Eng-C can reverse a pre-existing pathology and provide long-term therapeutic benefits, we treated 12-week-old G3Stg/*Gla*KO mice with either 5 × 10^10^ vg/kg or 2.5 × 10^11^ vg/kg of rAAV9-Eng-C (Study #2). Animals treated with a null vector at 2.5 × 10^11^ vg/kg and vehicle-treated wild type mice were used as controls. An additional group of untreated G3Stg/*Gla*KO mice was sacrificed at baseline to assess pre-treatment pathological changes. Treatment efficacy was analyzed over 18 weeks post dose. A dose-dependent increase in serum α-GAL activity was observed 2 weeks after rAAV9-Eng-C administration and was sustained throughout the duration of the study (Figure 5). The α-GAL protein levels were consistent with activity (Supplemental Table S5).

#### 3.3.2. rAAV9-Eng-C Treatment Cleared Substrate and Reversed Kidney Structural and Functional Pathology

**Figure 6:**
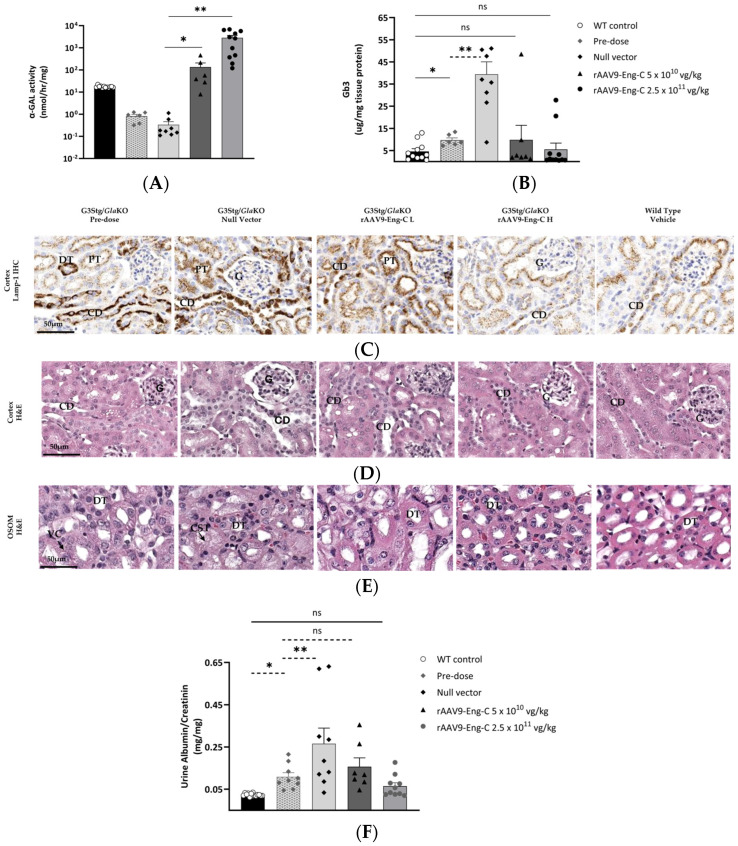
Reversal of renal pathology. Kidney α-GAL activity was measured in tissue homogenate using 4-MU-α-gal fluorescent substrate. Gb3 was quantified by LC/MS. (**A**) Dose-dependent increase in activity was observed in animals treated with rAAV9-Eng-C. (**B**) Gb3 reduction was proportional to dose, and the level of Gb3 in the rAAV9-Eng-C-treated groups was not statistically different from the level in the WT control. Activity of the rAAV9-Eng-C-treated groups was compared to the Null vector, and the Gb3 level was compared between WT control, Pre-dose, and rAAV9-Eng-C-treated groups, and between Pre-dose and Null vector groups using unpaired two-tailed T-tests in GraphPad Prism 10.2.1. ns > 0.05, * *p* < 0.05, ** *p* < 0.01; *n* = 6–12. (**C**) Kidney cortex immunohistochemistry with anti-LAMP1 antibodies. LAMP1 antibody detects lysosome-associated membrane protein 1, indicating an increase in the lysosomal compartment due to substrate accumulation evident at baseline. Collecting ducts and distal tubules were affected the most. Intensity of LAMP1 staining was completely normalized in mice treated with high dose of rAAV9-Eng-C. (**D**,**E**) Kidney cortex and outer strip of outer medulla H&E staining. Cytoplasmic vacuolization and luminal dilation were detected in G3Stg/*Gla*KO mice at baseline. Pathology continued to progress in Null vector control mice. Normal renal histology was observed in mice treated with a high dose of rAAV9-Eng-C. A low dose prevented further pathology progression. LAMP1 immunostaining (Brown). G, Glomeruli; CD, collecting duct; PT, proximal tubule; DT, distal tubule; CST, protein cast; VC, vacuole; OSOM, outer strip of outer medulla. (**F**) Urinary albumin and creatinine were measured using a COBAS C 311 analyzer. The albumin level was elevated at baseline and increased further by the end of the study in the Null vector group. In the high-dose rAAV9-Enz-C-treated group, pathology was reversed, and the albumin level was not statistically different from the WT control. Treatment with a low dose prevented albuminuria progression. Treated and control groups were compared to pre-treatment (dashed line) and WT control (solid line) groups using one-way ANOVA in GraphPad Prism 10.2.1. ns > 0.05, * *p* < 0.05, ** *p* < 0.005; *n* = 7–13.

Significant substrate accumulation and increased lysosomal burden were detected in kidneys of the 12-week-old G3Stg/*Gla*KO mice at baseline (Figure 6B,C). Consistent with findings in Fabry patients [30], the distal tubules and collecting ducts were affected the most, which led to pathological changes such as cytoplasmic vacuolization and luminal dilation observed at baseline (Figure 6D,E). Treatment with rAAV9-Eng-C resulted in a dose-proportional increase in α-GAL activity and a reduction in Gb3 levels and lysosomal burden (Figure 6A–C). α-GAL protein levels were consistent with activity (Figure 6A, Supplemental Table S5). The Lyso-Gb3 level was normalized in most animals in both dose groups (Supplemental Table S6). Progression of structural pathology was prevented in the low-dose group. In the high-dose group, pre-treatment pathology was reversed, and kidney structures were indistinguishable from normal ones upon histological examination. In contrast, kidney pathology continued to progress in mice treated with the null vector, leading to tubular atrophy, as evident by protein cast formation (Figure 6C–E). There was a strong to moderate but still statistically significant Spearman correlation between residual kidney Gb3 and kidney or serum α-Gal activity when a subset of samples from treated animals was analyzed, excluding samples where Gb3 was reduced below the WT level. Pearson correlation was not significant, indicating that the variables tend to consistently increase or decrease together, but the relationship is not linear, and the rate of change of the dependent variable Gb3 with the change of α-GAL level is not constant. Correlation between serum activity and kidney Gb3 was stronger than between kidney activity and kidney Gb3 (correlation coefficient of −0.69 vs. −0.94), potentially due to sampling. Gb3 accumulation in kidneys is cell type specific, with podocytes and distal tubular epithelia being significantly affected, while proximal tubular epithelia do not accumulate substrate. At the same time, by histological examination of treated animals’ kidneys, the highest α-GAL positivity can typically be detected in proximal tubules, where α-GAL cleared from the circulation is reabsorbed. Differences in the fraction of high α-GAL containing but unaffected by substrate accumulation of proximal and Gb3 loaded distal tubules in samples analyzed for activity and substrate for the same animal can add significant variability and complicate the correlation between tissue α-GAL activity and substrate. Therefore, serum α-GAL could be a better predictor of kidney efficacy (Supplemental Table S7).

Consistent with substrate accumulation and structural kidney damage, at baseline, G3Stg/*Gla*KO mice presented with a mild yet statistically significant increase in urinary albumin. By the end of the study, albuminuria significantly increased from baseline in the null vector-treated group, indicating progression of renal pathology. In the low-dose group, kidney function stabilized, and at the end of the study, the level of urinary albumin was not statistically different from baseline. Treatment with the higher dose reversed pre-treatment pathology and reduced urinary albumin to the WT level (Figure 6F).

#### 3.3.3. rAAV9-Eng-C Treatment Reversed Substrate Accumulation in Cardiomyocytes and Early Fibrotic Pathology in the Heart

**Figure 7:**
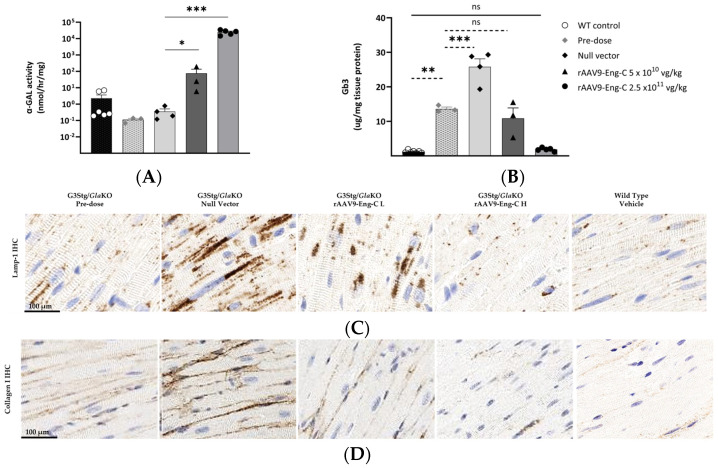
Reversal of cardiac pathology. Heart α-GAL activity was measured in tissue homogenate using 4-MU-α-gal fluorescent substrate. Gb3 was quantified by LC/MS. (**A**) Dose-dependent increase in activity was observed in animals treated with rAAV9-Eng-C. Due to the small number of animals per group, the increase did not achieve statistical significance in the low-dose group. (**B**) Gb3 accumulation was detected at baseline and was increased further by the end of the study. Gb3 reduction was proportional to dose, and the level of Gb3 in the rAAV9-Eng-C high-dose group was not statistically different from the level in the WT control. Treatment with a low-dose prevented further accumulation from the baseline. Activity of the rAAV9-Eng-C-treated groups was compared to Null vector + Pre-treatment (to increase *n*), and the Gb3 level was compared between WT control and Pre-dose and rAAV9-Eng-C-treated groups, and between the Pre-dose and Null vector groups using unpaired two-tailed T-tests in GraphPad Prism 10.2.1. ns > 0.05, * *p* < 0.05, ** *p* < 0.01, *** *p* < 0.001; *n* = 3–7. (**C**) Heart immunohistochemistry with anti-LAMP1 antibodies indicated an increase in the lysosomal compartment at baseline and a progressive increase in the Null vector control animals. Intensity of LAMP1 staining was completely normalized in mice treated with a high dose of rAAV9-Eng-C and did not increase from baseline in the low-dose group. (**D**) COL-I immunostaining was increased at baseline, and intensity was further increased in the control G3Stg/*Gla*KO mice. High-dose rAAV9-Eng-C treatment reversed baseline pathology and low-dose prevented progression.

Similar to the effect observed in kidneys, the substrate level in the hearts of the G3Stg/*Gla*KO mice was significantly elevated at baseline and increased further by the end of the study (16 weeks) in the G3Stg/*Gla*KO control group. The increase in collagen I (COL-I) immunostaining intensity was consistent with substrate accumulation, suggesting fibrosis initiation at baseline and progression in animals with α-GAL deficiency (Figure 7B–D). Administration of rAAV9-Eng-C resulted in a dose-dependent increase in α-GAL activity, leading to a robust reduction of Gb3 substrates in the low-dose group and near normalization (90% to 95% reduction) in the high-dose group (Figure 7A,B). Lysosomal (LAMP1) and fibrotic (COL-I) markers did not increase from the pre-dose level in the rAAV9-Eng-C low-dose group, indicating prevention of disease progression. High-dose treatment normalized these biomarkers to the wild type levels, efficiently reversing pre-existing pathological changes (Figure 7C,D).

α-GAL protein levels in the heart were consistent with activity (Figure 7A, Supplemental Table S5). Similar to Gb3, the lyso-Gb3 level was normalized in the high-dose group and was significantly reduced by the low-dose treatment (Supplemental Table S6). There was a good negative correlation between residual cardiac Gb3 levels and heart and serum α-Gal activity, but significance of the Spearman correlation was higher than Pearson, suggesting monotonic but not necessarily linear relationships (Supplemental Table S8). Although, since only half of the animals from each group were used for biochemical analysis, the sample number was small and likely affected *p*-values.

#### 3.3.4. Pre-Existing GI Pathology Was Reversed in rAAV9-Eng-C-Treated Dose Groups at Both Dose Levels

**Figure 8:**
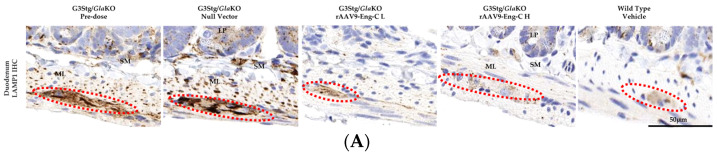

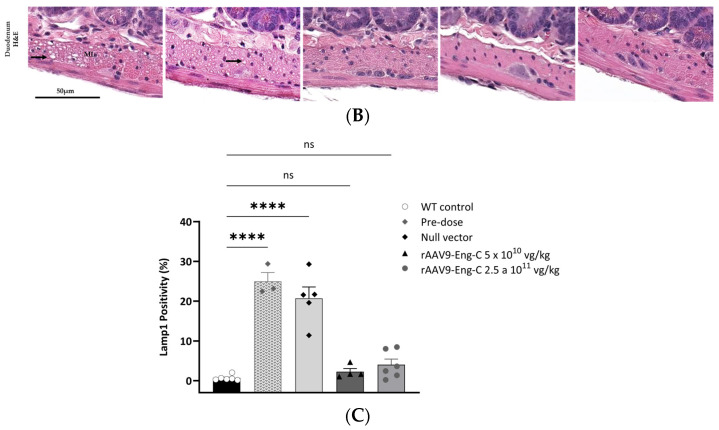
Reversal of GI pathology. Duodenum. (**A**) Increased LAMP1 immunostaining intensity (brown) was observed in the myenteric plexus ganglia (MPG), smooth muscles, submucosal layer, and lamina propria of the villi in the duodenum of the G3Stg/*Gla*KO mice at baseline. In mice treated with the Null vector, accumulation persisted through the end of the study. Treatment with rAAV9-Eng-C normalized LAMP1 intensity in both dose groups. (**B**) H&E staining. Cytoplasmic vacuolization was detected in the GI smooth muscles at baseline. Pathology was reversed in the rAAV9-Eng-C treated animals in both dose groups. Red dashed oval, MPG; ML, muscle layer; SM, submucosa; LP, lamina propria; black arrow, cytoplasmic vacuoles. (**C**) Quantitative biomarker image analysis was performed using HALO^®^ image analysis software. Positivity was calculated in the MPG (red circle) using the following formula: positivity (%) = positive area (pixels)/total analyzed area (pixels) × 100%. Maximum level of LAMP1 positivity was detected in duodenum at baseline, and the level did not change through the end of the study in the Null vector-treated control animals. At the end of the treatment, pathology was reversed, and LAMP1 positivity was not different from the WT controls. LAMP1 positivity in rAAV9-Eng-C- and Null vector-treated and pre-dose groups was compared to WT controls using one-way ANOVA in GraphPad Prism 10.2.1. ns > 0.05, **** *p* < 0.0001; *n* = 3–6.

The gastrointestinal (GI) symptoms in Fabry patients are thought to be due to neuropathic and myopathic changes, leading to abdominal pain, bloating, nausea, constipation, and diarrhea, with onset in early childhood [31,32]. Gb3 accumulation within enlarged ganglion cells of the myenteric plexus ganglia (MPG) and smooth muscle cells are the main drivers of GI disfunction [32]. Consistent with early GI pathology onset, MPG substrate accumulation in G3Stg/*Gla*KO mice, as evident by the increase in LAMP1 immunostaining of the lysosomes in duodenum and colon, already reached near maximum levels at baseline. This increase persisted in the Null vector control group through the end of the study (Figure 8A,C and Figure S5). Administration of both low and high doses of rAAV9-Eng-C reversed MPG and smooth muscle pathology, as measured by reduced LAMP1 and normalization of vacuolization on the H&E staining, with both being indistinguishable from the WT control at the end of the study. Reduction of LAMP1 positivity was also observed in the submucosal layer and lamina propria of the villi (Figure 8A–C).

There were moderate Pearson and Spearman correlations of serum α-GAL activity and MPZ LAMP1 positivity (coefficients of −0.70 and −0.74, respectively). Pearson correlation was not statistically significant; therefore, the relationship between serum α-GAL and MPZ substrate load was directional but not strictly linear (Supplemental Table S9).

#### 3.3.5. Prevention of Progression or Partial Reversal of Pre-Existing Structural PNS Pathology by rAAV9-Eng C Treatment Resulted in Recovery of Sensory Function

**Figure 9:**
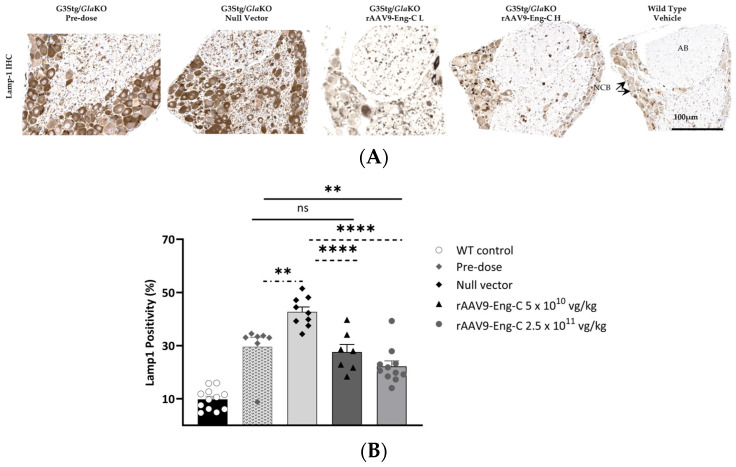
Normalization of lysosomal compartment in PNS of treated mice. (**A**) DRG immunostaining with anti-LAMP1 antibodies (brown). Increased LAMP1 immunostaining intensity was observed in the DRG neuronal bodies and axonal bundles in the pre-dose group. LAMP1 intensity was further increased in the Null vector control G3Stg/*Gla*KO mice. Treatment with rAAV9-Eng-C normalized LAMP1 levels in neuronal bodies and reduced in axonal bundles. NCB—neuronal cell bodies; AB—axonal bundle. (**B**) Quantitative biomarker image analysis was performed using HALO^®^ image analysis software. Positivity was calculated using the following formula: positivity (%) = positive area (pixels)/total analyzed area (pixels) × 100%. LAMP1 positivity was increased at baseline, and the level increased further in the Null vector-treated control animals at the end of the study. Baseline LAMP1 positivity was decreased in the high-dose rAAV9-Eng-C-treated group and stabilized in the low-dose group. The pre-dose group was compared to the WT control and rAAV9-Eng-C-treated groups (solid line), and the Null vector control was compared to rAAV9-Eng-C-treated groups (dashed line) using one-way ANOVA in GraphPad Prism 10.2.1. The pre-dose group was also compared to the Null vector control (dash-dotted line) using an unpaired two-tailed *t*-test in GraphPad Prism 10.2.1. ns > 0.05, ** *p* < 0.01, **** *p* < 0.0001; *n* = 6–11.

Histopathological findings in patients and animal models suggest that peripheral neuropathy in Fabry disease is due to substrate accumulation in dorsal root ganglia (DRG) neurons, associated nerve fibers, and supporting cells [33,34,35]. Pathology in PNS of G3Stg/*Gla*KO mice was consistent with that reported for Fabry patients. Significant substrate accumulation in DRG was detected at baseline, as indicated by an increase in LAMP1 immunoreactivity. Both neuronal cell bodies and axonal bundles were affected. Substrate accumulation increased further in control G3Stg/*Gla*KO mice treated with the Null vector. Treatment with rAAV9-Eng-C prevented pathology progression in a dose-dependent manner, and LAMP1 positivity was significantly lower in both dose groups at the end of the study when compared to the Null vector control. Strikingly, in animals treated with the high rAAV9-Eng-C dose, LAMP1 positivity was also significantly reduced from baseline, indicating clearance of pre-treatment substrate accumulation and normalization of lysosomal compartment. At the end of the study, LAMP1 positivity was normal or near normal in all neuronal bodies and reduced from baseline in axonal bundles (Figure 9A,B).

There was a good Spearman correlation between DRG LAMP1 positivity and serum α-GAL activity, with a correlation coefficient of −0.76 and *p* = 0.0002 (Supplemental Table S9). In severe Fabry patients, significant Gb3 accumulation was also found in the spinal cord [34]. In the G3Stg/*Gla*KO mice, we observed only a small increase in LAMP1 positivity in the nerve fibers of the posterior/dorsal root of the spinal cord at baseline, but pathology continued to progress in the Null vector control group. Clusters of unmyelinated axons appeared to be the most affected, which is consistent with the report on PNS Gb3 accumulation patterns in another Fabry mouse model *Gla*KO [33]. A slight increase in LAMP1 positivity was also observed in the dorsal horn of the spinal cord. Treatment with low doses of rAAV9-Eng-C prevented further progression and high doses reversed pre-existing pathology (Supplemental Figure S6).

**Figure 10:**
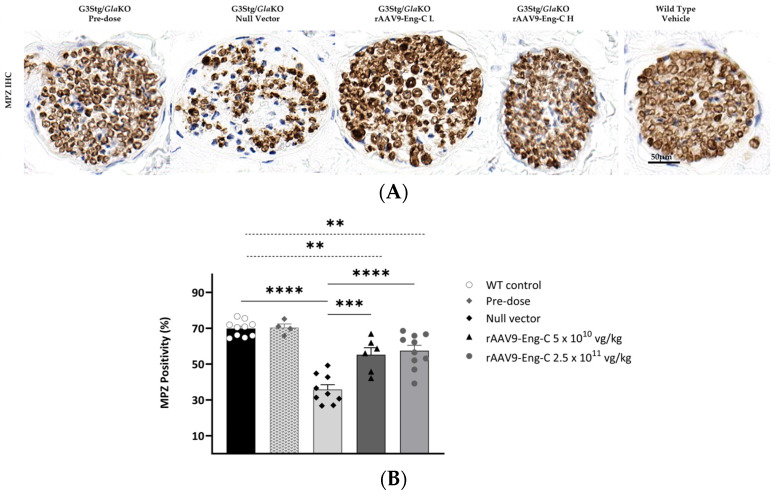
Small fiber preservation in dermis of G3Stg/*Gla*KO mice treated with rAAV9-Eng-C. (**A**) Paw dermis immunostaining with anti-MPZ antibodies (brown). MPZ staining was normal at baseline. By the end of the study, small fiber density was reduced in mice treated with the Null vector control, as evidenced by the decrease of MPZ-positive structures. Treatment with rAAV9-Eng-C preserved small fiber density. (**B**) Quantitative biomarker image analysis was performed using HALO^®^ image analysis software. Positivity was calculated using the following formula: positivity (%) = positive area (pixels)/total analyzed area (pixels) × 100%. MPZ positivity was significantly higher in rAAV9-Eng-C-treated groups than in the Null vector control but lower than in the WT control. The Null vector group was compared to WT control and rAAV9-Eng-C-treated groups (solid line) and the WT control was compared to rAAV9-Eng-C-treated groups (dashed line) using one-way ANOVA in GraphPad Prism 10.2.1. ns > 0.05, ** *p* < 0.01, *** *p* < 0.001, **** *p* < 0.0001; *n* = 6–11.

Another hallmark of Fabry disease is the reduction in small unmyelinated and thinly myelinated dermal nerve fiber density (DNFD). To assess DNFD in G3Stg/*Gla*KO mice and the effect of the rAAV9-Eng-C treatment, we used a myelin protein zero (MPZ) antibody for paw dermis immunostaining (Figure 10A). Histological sections were scanned, and MPZ positivity was quantified using HALO^®^ image analysis software (Figure 10B). There was no loss of thinly myelinated fibers in the paws of the G3Stg/*Gla*KO mice at baseline compared to control mice, as indicated by a normal MPZ positivity level. At the end of the study, G3Stg/*Gla*KO mice treated with rAAV9-Eng-C had more MPZ-positive small fibers than Null vector-treated mice but less than WT mice (Figure 10A,B).

**Figure 11:**
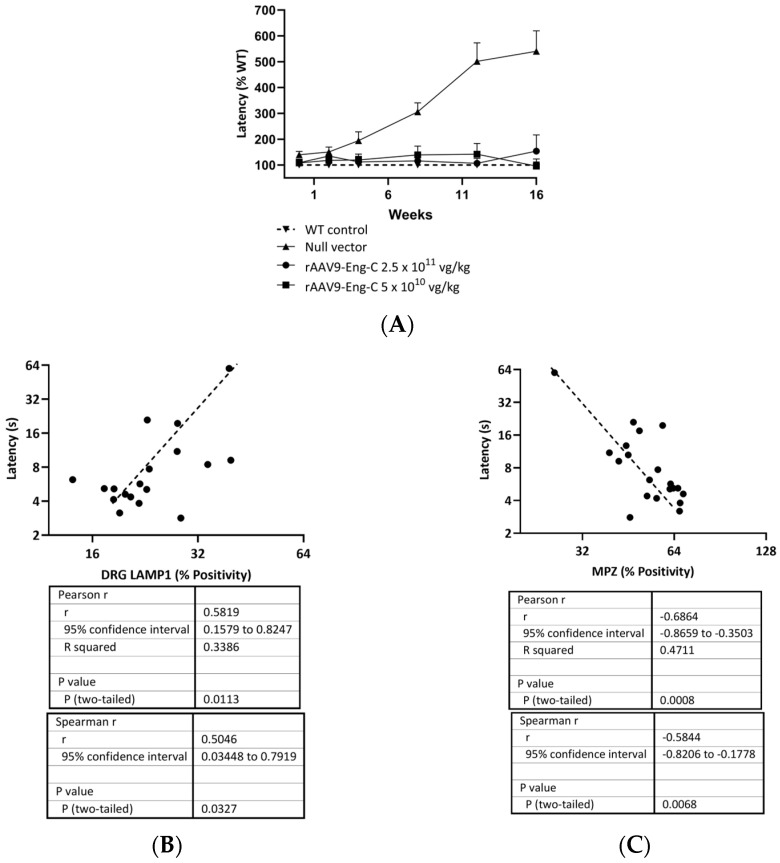
Hot-plate latency. (**A**) Progressive increase in hot-plate latency was observed in the G3Stg/*Gla*KO mice treated with the Null vector control. Treatment with rAAV9-Eng-C prevented sensory deficits in both dose groups. (**B**) Correlation of hot-plate latency with DRG LAMP1 positivity. (**C**) Correlation of hot-plate latency with hind paw MPZ positivity. Correlation between latency and DRG LAMP1 or MPZ positivity was assessed using GraphPad Prism 10.2.1 Spearman and Pearson functions. Data for animals treated with rAAV9-Eng-C and Null were included in the analysis. A dashed line represented a regression line from the indicated correlation.

Efficacy of the rAAV9-Eng-C treatment in preserving sensory function was assessed in the hotplate latency test at baseline and 2, 4, 8, 12, and 16 weeks after dosing. Sensitivity to heat in the G3Stg/*Gla*KO mouse groups was slightly compromised at baseline and/or week 2 when compared to the WT control (Supplemental Table S10). Sensory deficit as measured by hot plate sensitivity worsened further with age in mice treated with the Null vector. Treatment with rAAV9-Eng-C normalized thermal sensitivity and hotplate latency in both rAAV9-Eng-C-treated groups and was not different from the WT control at the end of the study (Figure 11A, Supplemental Table S10). Hot plate latency at 16 weeks correlated positively with DRG lysosomal burden and negatively with small fiber density. (Figure 11B,C). Non-nociceptive factors such as body weight, habituation, and repeated testing are known to influence hot-plate latency and are strong contributory factors for variability in this test. Therefore, moderate correlation coefficients are not surprising. Nevertheless, it is worth noting that both Pearson and Spearman correlations were statistically significant, denoting linearity and monotony of the relationships between latency and DRG pathology and latency and small fiber density.

### 3.4. Therapeutic α-GAL A Levels Required for Efficacy in Mitigating Different Pathological Manifestations

**Figure 12:**
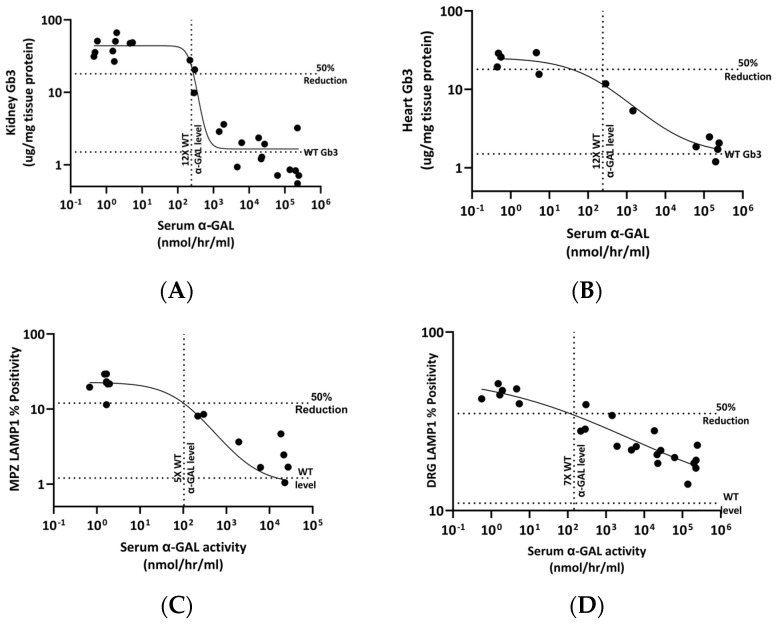
Serum α-GAL concentration and residual substrate level were fitted into a nonlinear four parameter logistic (4PL) regression with variable slope using GraphPad Prism 10.2.1. The R^2^ values were the following: kidney = 0.88, heart = 0.9, MPZ = 0.85, DRG = 0.88. The purpose of the curve fitting was to predict plasma α-GAL A values for 50% substrate reduction expressed as Gb3 concentration in kidney (**A**) and heart (**B**) tissue homogenate, and myenteric plexus ganglia (**C**) and DRG (**D**) LAMP1 positivity.

The range of plasma and tissue α-GAL levels in mice treated with two dose levels of rAAV9-Eng-C varied from just above normal to supraphysiological >1000-fold of mean normal. Substrate reduction was dose proportional and inversely correlated with plasma α-Gal levels. Predicted α-GAL levels for minimal efficacy (defined as 50% reduction in Gb3) were 12× the mean normal α-Gal levels for kidneys and heart, 5× for GI, and 7× for DRG (Figure 12A–D).

**Figure 13:**
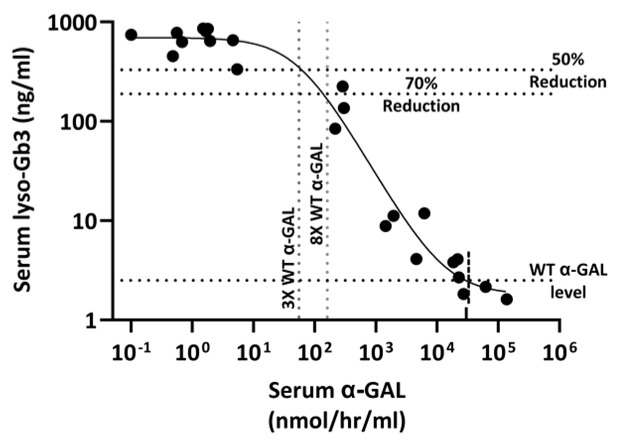
Serum α-GAL concentration and residual lyso-Gb3 level were fitted into a nonlinear four parameter logistic (4PL) regression with variable slope using GraphPad Prism 10.2.1. R^2^ = 0.9. The purpose of the curve fitting was to predict plasma α-GAL values for different levels of lyso-Gb3 reduction.

Recently, serum lyso-Gb3 became a preferred biomarker for therapeutic monitoring of Fabry patients [36]. Nonlinear regression analysis indicates that 8-fold of normal serum α-GAL levels would be sufficient to reduce serum lyso-Gb3 by 70% (Figure 13). At the same time, complete clearance could require >100-fold of normal, which is consistent with nonlinear relationships between α-GAL levels and Gb3 reduction (significant Spearman but not Pearson correlation, Supplemental Table S11)

### 3.5. Administration of rAAV9-Eng-C to the NHP Resulted in a Dose-Dependent Suprafisiological Serum α-GAL Level

The pharmacokinetic profile of α-GAL A in plasma was evaluated in a 3-month NHP study. Animals were dosed with rAAV9-Eng-C 6.25 × 10^12^ vg/kg (Group 2: 2 females, 1 male) and 3 × 10^13^ vg/kg (Group 3: 4 females, 3 males). Two animals were injected with buffer and used as a control. Cynomolgus monkeys for the study were selected based on the negative results of pre-existing neutralizing anti-AAV9 antibodies 4 weeks prior to study initiation. On study Day 1, prior to dosing, a pre-existing anti-AAV9 neutralizing antibody screen was conducted once more. High total (Tab) and neutralizing (Nab) anti-AAV9 antibody titers were detected in two animals in Group 3. Two more animals, one from each group, had total antibody titers of 400. Overall, only one monkey had anti-AAV9 NAb at the preferred cut-off titer of 1:5. Similar to what was demonstrated in other studies [37], retrospective analysis of this study showed that there was a correlation between the anti-capsid antibodies and Eng-C transgene protein in plasma samples (Table S12).

**Figure 14:**
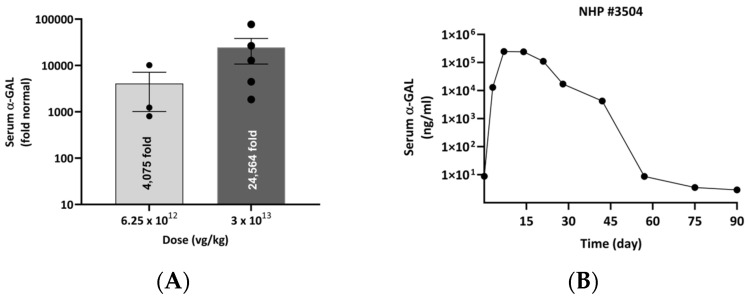
Serum a-GAL. (**A**) Fold of normal at 2 weeks (**B**) Time course for NHP #3504 treated with 3 × 10^13^ vg/kg of rAAV9-Eng-C. rAAV9-Eng-C administration was well tolerated in all animals, and in-life assessments were within normal parameters, including safety pharmacology endpoints. A data point was represented as a closed circle (•).

Following a single IV infusion, the mean Cmax values for rAAV9-Eng-C increased dose proportionally and peaked at about 2 weeks after dosing. A 5-fold increase in the rAAV9-Eng-C dose resulted in an approximate 6.0-fold increase in mean Cmax. As expected, dose-adjusted plasma α-GAL levels in NHPs were ~2 logs lower than in mice due to species differences in translation efficiency [38]. The presence of the anti-Eng-C antibodies was detected in most animals by Day 21 or Day 28, likely contributing to a quick loss of Eng-C expression. Nevertheless, a supraphysiological level of >4000-fold of normal/endogenous a-GAL was achieved, with the lowest tested dose of 6.25 × 10^12^ vg/kg (Figure 14A), indicating that a low rAAV9-Eng-C dose in the 10^12^ vg/kg range could potentially be sufficient for complete substrate clearance in most affected tissues. In an anti-AAV9 antibody negative at baseline monkey that did not develop anti-Eng-C until Day 42, the maximum serum Eng-C level was the highest and maintained the longest, with the decline coinciding with the rise of the anti-Eng-C antibodies (Figure 14B, Table S12).

### 3.6. rAAV9-Eng-C GT Was Well Tolerated in WT Mice in a Non-GLP Dose Ranging Study

**Figure 15:**
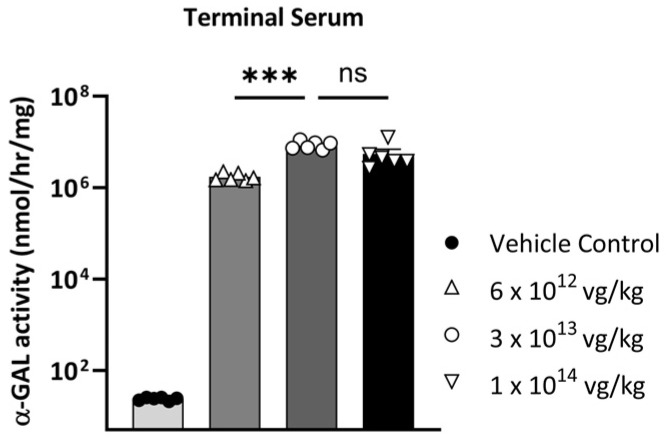
Toxicokinetic Profile. Serum α-GAL activity between groups was compared using one-way ANOVA in GraphPad Prism 10.2.1. ns > 0.05, *** *p* < 0.001; *n* = 6. Increase between low- (6 × 10^12^ vg/kg) and mid-dose (3 × 10^13^ vg/kg) groups was significant and dose proportional. There was no further increase in α-GAL activity in the high-dose group, and the levels in the mid- and high-dose groups were not statistically different.

A 6-week non-GLP dose range finding study was conducted to determine the maximum tolerated dose (MTD) and toxicokinetics of rAAV9-Eng-C GT when administered via a single intravenous tail vein injection to 7–9 week old male C57BL/6 mice. Male mice (6/dose cohort) were assigned to four dose cohorts: vehicle buffer, 6 × 10^12^ vg/kg, 3 × 10^13^ vg/kg, and 1 × 10^14^ vg/kg. All animals survived to scheduled necropsy on Day 43, and no rAAV9-Eng-C-related findings were noted on clinical observations, body weights, body weight gains, food consumption, and macroscopically. Non-adverse clinical pathology findings in red blood cell parameters and liver transaminases were observed at all dose levels in a dose-dependent manner. Serum α-GAL activity was measured at a single time point for all dose levels at 43 days post-dose rAAV9-Eng-C. Mean serum levels were determined to be 1.7 × 10^6^, 8.6 × 10^6^, and 5.5 × 10^6^ nmol/hr/mL in the low-, mid-, and high- dose groups, respectively, resulting in supraphysiological levels of circulating serum α-GAL levels compared to vehicle-treated animals (Figure 15).

## 4. Discussion

Gene transfer as a therapy has been explored for more than 50 years [39]. It is a natural fit for treating monogenic diseases such as Fabry. Two decades ago, scientists at Genzyme demonstrated the efficacy of AAV-based liver directed gene therapy (GT) in the Fabry mouse model. It was established that only a small proportion of the protein produced from a GT vector is taken up by distant target tissues. Unfortunately, further studies in nonhuman primates revealed an unexpected drop in efficiency of liver α-GAL production and secretion in higher mammals due to the lower transcription efficiency, and it became evident that achieving the 1 µg/mL α-GAL in circulation predicted to be required for therapeutic efficacy was not feasible. Consequently, the GT candidate was not advanced to clinical trials [38,40,41,42]. The requirement of a high supraphysiological level of serum α-GAL for GT efficacy through cross-correction has been confirmed by a number of similar studies that followed [43,44,45,46,47]. Over time, with better designs, efficiency of transgene expression from AAV GT vectors improved, and several Fabry AAV GTs advanced into clinical trials. But, so far, achieving high supraphysiological levels required in mouse studies for complete substrate clearance has not been possible.

Serum α-GAL in the first three patients treated with FLT190 (Freeline Therapeutics, London, UK, NCT 04040049) was below normal. Worsening of symptoms was reported for one of the patients, two patients showed signs of mild transient myocarditis, and the trial was discontinued [48]. In patients treated with ST-920 (Sangamo Therapeutics, NCT 04046224) and 4D-310 (4D Molecular Therapeutics, Emeryville, CA, USA, NCT 04519749), serum α-GAL levels were variable, without an obvious dose response, and significantly lower than the Genzyme predicted ultimate efficacious level. Nevertheless, patients were able to discontinue ERT, and some encouraging preliminary efficacy was reported for both candidates [15,16,49]. Recently, the first patient was dosed with another liver directed AAV GT candidate, AMT-191 (UniQure Biopharma, Amsterdam, The Netherlands, NCT06270316) [50].

Despite this progress, concerns about inefficient delivery, immunogenicity, high dose toxicity, and limited tissue exposure persist. All current GTs are based on the native *GLA* sequences and require more than 13 vg/kg doses to achieve therapeutic levels. The root of this problem potentially lies in the nature of the native α-GAL enzyme. α-GAL is an intracellular enzyme with low stability in circulation and thus poorly suited to serve as a therapeutic, either as an ERT or delivered through gene therapy. In view of the potential limitations of the first generation Fabry GTs delivering the native α-GAL enzyme, we developed a novel candidate with an engineered *GLA* transgene. We focused on the biology of the disease and desired outcome of the treatment to tailor α-GAL properties to the specific needs of Fabry patients. A high expression level and stability are key requirements for optimizing GT where cross-correction is the main mechanism driving efficacy. The Eng-C variant selected for GT development was highly stable in serum and at lysosomal pH [18]. In addition, Eng-C had greatly reduced predicted immunogenicity. This variant was identified by screening 12,000 α-GAL variants through multiple rounds of directed evolution, selecting for enhanced stability in lysosomal and blood pH, increased stability in serum, and increased activity in cells in the earlier rounds of evolution, followed by lower immunogenicity in the later rounds. The Major Histocompatibility complex (MHC) system known as the human leukocyte antigen (HLA) plays an essential role in immune responses. MHC Class II receptors are found on the professional antigen-presenting cells and are responsible for displaying endogenous peptides to initiate immune responses to foreign proteins through CD4+ T cell activation. Immunogenicity of biologics represents a significant hurdle in the continuing therapy of patients with genetic diseases where a biologic is administered to restore function of a missing protein and is often treated by the immune system as foreign. One of the goals for the α-GAL variant selection was reduction of immunogenicity by minimizing the number and potency of MHC II-binding epitopes without activity loss. All strong epitopes responsible for 50% of the immunogenicity were eliminated in the Eng-C α-GAL variant, and the number of moderate epitopes was significantly reduced. In silico prediction was consistent with reduced Eng-C cross-reactivity with pre-existing anti- α-GAL antibodies in Fabry patients’ serum.

Improved functional properties of Eng-C translated into better PK and tissue exposure, as well as better substrate clearance by rAAV9-Eng-C as compared to rAAV9-h*GLA*, expressing the native *GLA* sequence. Vector genome and mRNA levels were not statistically different for corresponding doses of rAAV9-Eng-C and rAAV9-h*GLA*, but serum and tissue α-GAL levels were 10- to 55-fold higher for rAAV9-Eng-C, except for the liver, where the increase over rAAV9-h*GLA* was lower potentially due to saturation of lysosomal capacity, resulting in a larger secreted fraction. Overall, a five-times lower dose of rAAV9-Eng-C had same or better tissue α-GAL exposure and substrate reduction efficacy than rAAV9-h*GLA*. We did not evaluate development of anti-transgene antibodies in mouse studies. But we found that α-GAL serum levels were not affected by any potential antibodies that could have developed. α-GAL levels were sustained through the duration of all mouse studies conducted for 1, 4 or 8 months (Figure 5 and Figures S3 and S7).

To assess the efficacy of rAAV9-Eng-C in preventing disease progression and reversing the pre-existing pathology, we used a more severe G3Stg/*Gla*KO mouse model that develops some abnormalities analogous to Fabry patients [22]. We found that similar to Fabry patients [30], at baseline, G3Stg/*Gla*KO mice had significant Gb3 accumulation in kidney distal tubules and collecting ducts, leading to cytoplasmic vacuolization and luminal enlargement. Continuing substrate accumulation was observed in the Null vector-treated mice, leading to pathology progression resulting in tubular atrophy, as evidenced by the presence of the protein casts. Worsening of the structural pathology was halted in the low-dose rAAV9-Eng-C group and reversed in the high-dose group 4 months after treatment initiation. The effect of rAAV9-Eng-C on kidney function was consistent with efficacy in reversing and preventing kidney damage. A mild increase in urinary albumin at baseline was reversed by treatment with the higher dose and stabilized with the lower dose. It is noteworthy that even though treatment with rAAV9-Eng-C completely cleared the substrate in most animals, by the end of the study in both dose groups, only the higher dose reversed a pre-existing pathology. This could be due to the rate of substrate clearance. In a shorter 1-month study (Study #1), we observed complete kidney substrate normalization in all but one animal in the high-dose group, whereas in the low-dose group, while not statistically different, the substrate level was still slightly above the mean normal in half of the animals (Figure 4C). Therefore, early treatment initiation and rapid substrate clearance could mean the difference between stabilization of remaining function and reversal of pre-existing pathology with complete normalization of function. Reduction in collagen immunostaining in kidneys was consistent with substrate reduction. In the high-dose group, baseline pathology was reversed, and the low dose prevented from further progression.

Fabry GI pathology is caused by substrate accumulation in myenteric ganglia neurons, which form part of the PNS, smooth muscles, and other cell types. GI substrate deposits appear to be the most sensitive to treatment. Complete or near complete clearance of baseline substrate was achieved in the GI of rAAV9-Eng-C-treated animals in both dose groups. This result is consistent with some improvement of gastrointestinal symptoms, such as diarrhea and abdominal pain, that has been reported in some Fabry patients relatively shortly after the initiation of ERT and correlated with Gb3 clearance in enterocytes [50]. In addition, rapid worsening of GI symptoms after ERT dose reduction was also reported [51,52]. Therefore, GI symptoms could be a quick proof of efficacy for a new therapy such as GT.

In addition to GI, PNS pathology in Fabry is manifested by significant substrate accumulation in DRG, resulting in small fiber neuropathy and sensory deficiency. In G3Stg/*Gla*KO mice, DRG LAMP1 immunostaining was already significantly higher than in WT controls at baseline. But paw small fiber density was not affected, and thermal sensitivity was only slightly increased in some of the mice. This is consistent with reports for Fabry patients where PNS pathology could be limited to DRG, and small fiber density is unaffected [53,54]. Treatment with rAAV9-Eng-C normalized thermal sensitivity even though the DRG substrate level was not fully normalized, and small fiber loss was not completely prevented. Despite this, DRG neuronal clearance was uniform, unlike the earlier reported results for rAAV9-h*GLA* treatment where some neuronal subtypes were resistant to treatment [17]. We found a positive correlation between DRG substrate load or small fiber density and thermal sensitivity. There are no quantitative patient data on the effect of the DRG substrate level on peripheral neuropathy in Fabry patients. Correlation between small fiber density and peripheral neuropathic manifestations in Fabry patients is generally not observed except for within cases of neuropathy in patients with normal kidney function [54]. This is consistent with our findings. Even though we observed some albuminuria in G3Stg/*Gla*KO mice, normal serum BUN (Supplemental Figure S8) suggests that kidney function was still unaffected.

The *Gla*KO mouse model, which is typically used to test the efficacy of Fabry therapeutics, does not accumulate substrate in cardiomyocytes known to be significantly affected in Fabry patients. In pre-clinical studies, recombinant human α-GAL was more efficient in clearing substrate in the heart than in kidneys. There, the results did not translate into efficacy in patients, who accumulate much higher levels of substrate in cardiomyocytes [55,56]. The *Gla*KO model was also used in pre-clinical studies for AAV GT therapies [46,47,50]. G3Stg/*Gla*KO mice are characterized by higher substrate accumulation, especially in the heart, where it is detected in cardiomyocytes. rAAV9-Eng-C treatment was efficient in renal and cardiac substrate clearance. It is noteworthy that using a similar approach to the one employed by Jeyakumar et al. [46] for FLT-190 Fabry GT tested in *Gla*KO mice, the predicted therapeutic α-GAL level for 50% Gb3 reduction in G3Stg/*Gla*KO mice was higher. The fold increase in predicted dose also corresponded to the tissue specific increase in substrate accumulation in the G3Stg/*Gla*KO model. For example, the level of serum α-GAL predicted to reduce cardiac Gb3 by 50% in our study was ~25-fold higher than predicted for FLT-190, consistent with 15-fold higher cardiac Gb3 levels in the G3Stg/*Gla*KO mice.

Analyses of our data suggest that the predicted α-GAL levels required for minimal efficacy (defined as 50% reduction in Gb3) varied between tissues and ranged from 5× to 12× normal. Recently, preliminary positive results were reported for a ST-920 Fabry gene therapy trial [15]. In three naïve patients with elevated serum lyso-Gb3 at baseline, treatment with ST-920 resulted in 2.5-, 3.5-, and 13-fold of normal increase in serum α-GAL activity, leading to a 55% to 70% of substrate reduction 4 months after dosing. This level was higher than in patients on ERT. There was no further substrate reduction over >1 year of additional observations. The largest reduction was observed in a patient with a higher baseline level, and the higher α-GAL activity increase was potentially more efficient, but more data are needed to make any conclusions. Sangamo clinical data are consistent with efficacy prediction using data from our mouse study. Nonlinear regression analysis indicates that an 8-fold of normal serum α-GAL level would be sufficient to reduce serum lyso-Gb3 by 70%, but complete clearance might require >100-fold of normal.

Studies in monkeys further confirmed that rAAV9-Eng-C therapy can deliver supraphysiological α-GAL levels at relatively low doses in a higher species. Although, due to post-COVID-19 challenges in NHP availability, it was not possible to source sufficient number of animals without pre-existing anti-AVV9 antibodies. Therefore, transduction efficacy was likely reduced in most monkeys, as reported by others [37]. We also observed an increase of anti-rAAV9 neutralizing antibodies approximately 14 days post-administration (Table S13). In addition, the loss of α-GAL expression coincided with the development of anti-Eng-C α-GAL antibodies. Strong immune response to human proteins has been reported in NHP AAV GT studies, especially when the transgene expression level is high, and no immunosuppressants were administered to NHP prior to dosing. Therefore, the loss of α-GAL expression in our study was not unexpected [57]. Nevertheless, serum α-GAL Cmax was > 4000-fold of mean normal in the low-dose group. This further supports the potential to achieve clinical efficacy in Fabry patients with a low-dose treatment. High systemic levels are not only important for efficient initial substrate clearance but are also necessary for long-term durability, as there is a likelihood of decrease in liver-produced transgene product levels due to hepatocyte turn-over and episomal loss reported for other gene therapies [58]. The safety and long-term durability of a gene therapy could be a decisive factor in patient adoption in a disease such as Fabry where there are established treatments, and patients’ risk tolerance is low [59]. In both NHP and non-GLP dose ranging mouse safety studies, in-life observations indicated that tested doses were well tolerated, which is consistent with nonclinical findings for other IV administered AAV-based gene therapies [36,42].

Overall, we found that rAAV9-Eng-C can deliver high supraphysiological levels of α-GAL at a low dose and could realize the true transformative potential of gene therapy in Fabry disease.

## 5. Conclusions

We found that treatment with rAAV9-Eng-C gene therapy delivering a *GLA* variant with improved therapeutic characteristics was significantly more efficient than gene therapies with the native *GLA* sequence. Baseline pathological changes in tissues of the G3Stg/*Gla*KO mouse model were either completely reversed or stabilized in a dose-dependent manner. Here, for the first time, we demonstrated that initial signs of fibrosis such as cardiac collagen deposits and renal tubular atrophy can be reversed. Our findings provide new insights into the feasibility of early pathology reversal in Fabry patients and confirm the requirement of supraphysiological levels of α-GAL to achieve optimal efficacy. Using an engineered α-GAL variant with improved stability could allow the achievement of target α-GAL levels with a low AAV GT dose, promising better safety.

## Data Availability

Data are available in the Supplementary Tables.

## Supplementary Materials

Supporting figures and tables can be downloaded at https://www.mdpi.com/article/10.3390/biomedicines13030577/s1, Figure S1: In-vitro characterization; Figure S2: Vector genome and mRNA level in the liver, heart and kidney; Figure S3: Serum α-GAL activity overtime; Figure S4: Correlation of serum α-GAL activity and liver vector genome (VG) copy number with tissue and serum α-GAL activity; Figure S5: Immunostaining with anti-LAMP1 antibodies; Figure S6: Immunostaining with anti-LAMP1 antibodies; Figure S7: Serum α-GAL activity overtime; Figure S8: Blood Urea Nitrogen (BUN) in the pre-dose and terminal serum samples; Table S1: α-GAL activity fold of mean normal and fold increase in rAAV9-Eng-C treated over rAAV9-hGLA treated animals (Ratio of rAAV9-Eng-C to rAAV9-hGLA; Table S2: Serum and tissue α-GAL activity correlation; Table S3: VG and α-GAL activity correlation; Table S4: Serum and tissue lyso-Gb3 level; Table S5: Serum and tissue α-GAL protein; Table S6: Serum and tissue lyso-Gb3; Table S7: Kidney and serum α-GAL activity and kidney Gb3 correlation; Table S8: Heart and serum α-GAL activity and heart Gb3 correlation; Table S9: Serum α-GAL activity and MPZ and DRG LAMP1 positivity correlation; Table S10: Hot plate latency test results; Table S11: Serum α-GAL activity and serum lyso-Gb3 correlation; Table S12: α-GAL concentration at 2 weeks, anti-AAV9 and anti-Eng-C variant in NHP serum at indicated day after treatment administration; Table S13: Anti-rAAV9 antibodies titers in the NHP study.

## Abbreviations

The following abbreviations are used in the manuscript:

AAV	Adeno-associated virus
ADA	anti-drug antibody
α-GAL	alpha galactosidase A
BUN	blood urea nitrogen
COL-1	Collagen I
DRG	dorsal root ganglion
DNFD	dermal nerve fiber density
ERT	Enzyme replacement therapy
ESRD	end stage renal disease
*Gla*KO	alpha galactosidase A knock out
Gb3	Globotriaosylceramide
GT	gene therapy
G3Stg/*Gla*KO	transgenic Gb3 synthase alpha galactosidase A knock out
GI	gastrointestinal
IEDB	immune epitope database
LAMP-1	lysosomal associated membrane protein 1
LVH	left ventricular hypertrophy
MPG	myenteric plexus ganglia
MTD	maximum tolerated dose
Nab	neutralizing antibody
NHP	non-human primate
PCT	pharmacological chaperone therapy
PNS	peripheral nervous system
rAAV	recombinant adeno associated virus
rh α-GAL A	recombinant human alpha galactosidase A
SMM align	stabilization matrix alignment method
tAb ADA	total anti-drug antibody
