# Supplementary material for: Reversing Pathology in an Aggravated Fabry Mouse Model Using Low-Dose Engineered Human Alpha-Galactosidase A AAV Gene Therapy
Source: Biomedicines. 2025 Feb 25;13(3):577. doi: 10.3390/biomedicines13030577 (PMC11940569; doi:10.3390/biomedicines13030577)
Supplement: Supplementary file 1 [file biomedicines-13-00577-s001.zip › biomedicines-3454907-Supplementary Materials.pdf]

biomedicines

MDPI

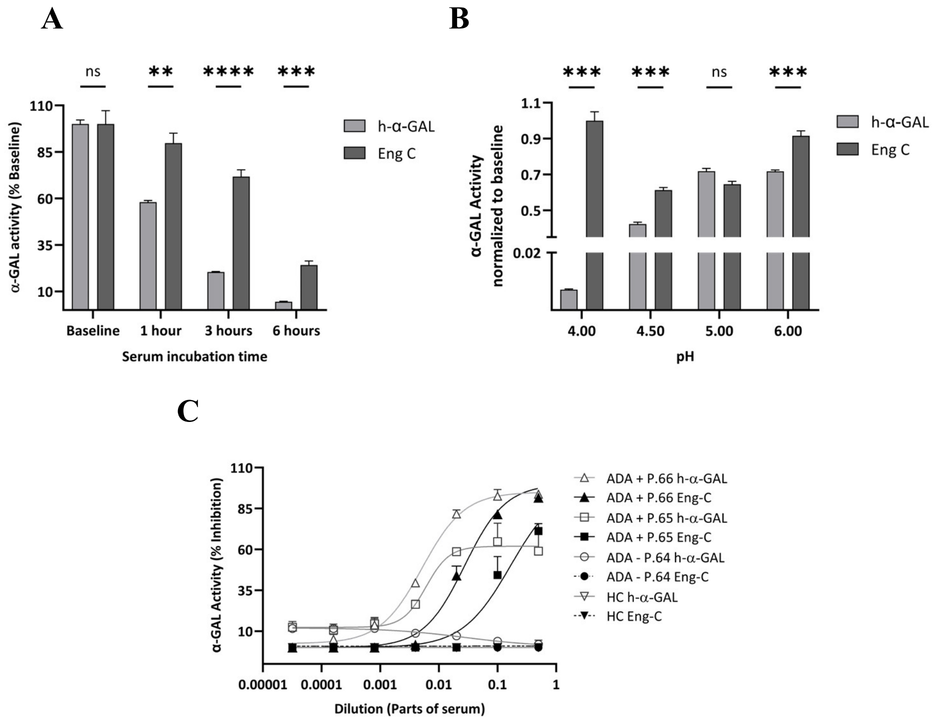

Figure S1. In-vitro characterization. (A) Serum stability was measured as the remaining activity of the enzymes analyzed using 4-MU-α-gal assay after indicated incubation time. The results are expressed as the percentage of the initial enzymatic activity at the beginning of the experiment. (B) Stability under lysosomal-like condition was measured as the remaining activity of the enzymes analyzed using 4-MU-α-gal assay after 24 hours incubation under indicated pH. Results were normalized to initial activity. (A, B) Data were analyzed using multiple T-test in GraphPad Prism 10.2.1, ns > 0.05, **p < 0.01, ***p < 0.001; n=4. (C) Cross-reactivity with pre-existing anti-α-GAL antibodies. Anti-drug antibodies (ADA) positivity in Fabry patient serum was established using ELISA. Samples #65 and #66 were positive and #64 was negative (data not shown). To test inhibition of enzymatic activity, native α-GAL and Eng-C proteins were pre-incubated with serially diluted patients' and healthy control (HC) serum samples (from 1:2 to 1:31,250) and activity was measured using 4-MU-α-gal fluorescent substrate. Healthy volunteers' pooled serum sample was used as a 100% activity for each dilution and to establish cut-off dilution of matrix effect. At 1:50 dilution sample #65 did not inhibit Eng-C activity while native α-GAL was inhibited by 60%. Sample #66 did not have any effect on Eng-C activity at 1:250 dilution while native α-GAL was inhibited by 40%. ADA negative serum did not have significant effect on activity of either protein, although, small (<10%), dilution independent inhibition of native α-GAL was observed.

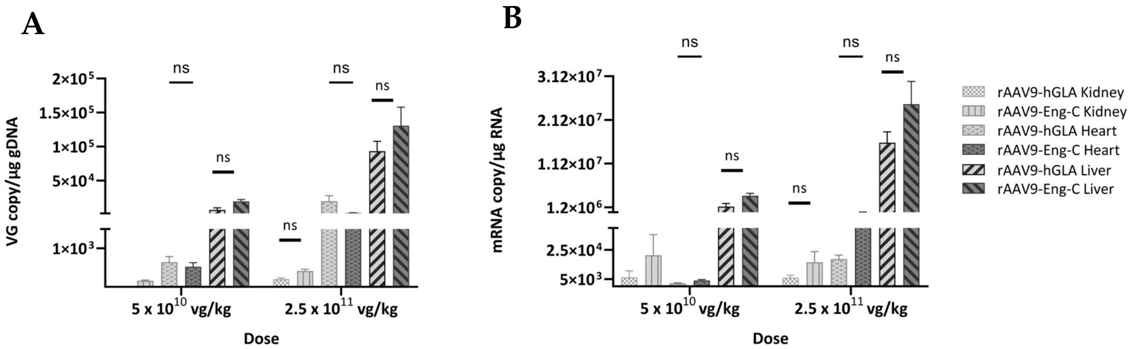

C

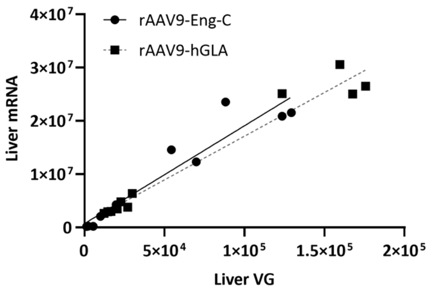

	VG vs rAAV9--Eng-C mRNA	VG vs rAAV9-hGLA mRNA
Pearson r		
r	0.9531	0.9805
95% confidence interval	0.8250 to 0.9881	0.9242 to 0.9951
R squared	0.9085	0.9614
P value		
P (two-tailed)	<0.0001	<0.0001

	rAAV9-Eng-C mRNA	rAAV9-hGLA mRNA
Best-fit values		
Slope	183.7	163.9

The difference between the slopes is not significant, p=0.374

Figure S2. Vector genome and mRNA level in the liver, heart and kidney. (A) Vector genome copy number per µg of genomic DNA. (B) mRNA copy number per ug of mRNA. VG and mRNA copy numbers were compared between corresponding tissues and dose groups of rAAV9-Eng-C and rAAV9-h*GLA* treated animals. Statistical analysis was performed using multiple unpaired t-test in GraphPad Prism 10.2.1, ns > 0.05; n= 5 – 6. (C) Correlation between VG and mRNA copy number in the liver. GraphPad Prism 10.2.1 Pearson correlation function was used for analysis. Differences in fit was assessed using Line function in GraphPad Prism 10.2.1.

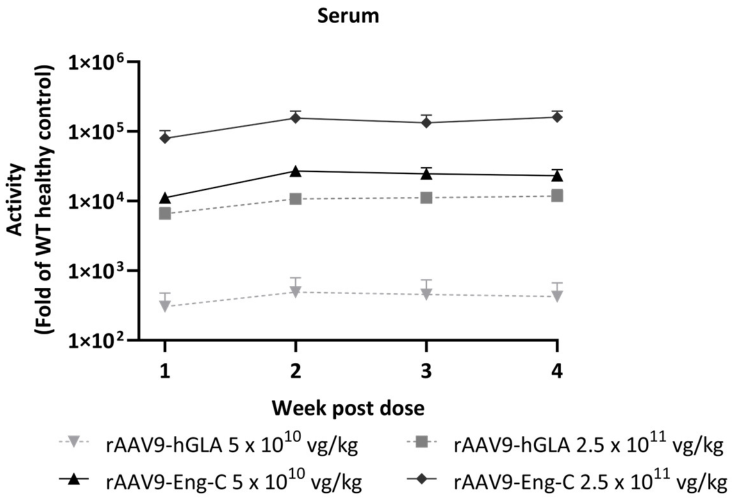

Figure S3. Serum α-GAL activity overtime. Serum samples were collected at 1, 2, 3 and 4 weeks after dosing. Activity was measured using 4-MU-α-gal fluorescent substrate. N = 7 to 12 per group/per timepoint

Construct	rAAV9-Eng-C (fold of normal)		rAAV9-hGLA (fold of normal)		Ratio of rAAV9-Eng-C to rAAV9-hGLA	
Dose / Tissue	5 x 10^{10} vg/kg	2.5 x 10^{11} vg/kg	5 x 10^{10} vg/kg	2.5 x 10^{11} vg/kg	5 x 10^{10} vg/kg	2.5 x 10^{11} vg/kg
**Serum**	2179	15038	39.8	1030	55	15
**Liver**	127	946	11.8	458	11	2
**Kidney**	26.7	288	1.43	31.2	19	9
**Heart**	130	1614	12	247	11	6.5

Table S1. α-GAL activity fold of mean normal and fold increase in rAAV9-Eng-C treated over rAAV9-h*GLA* treated animals (Ratio of rAAV9-Eng-C to rAAV9-h*GLA*).

A

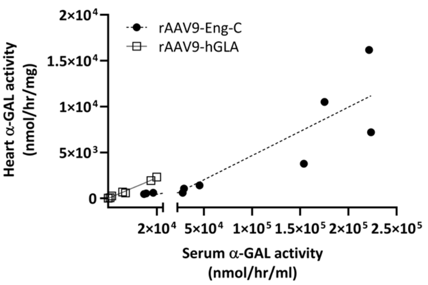

B

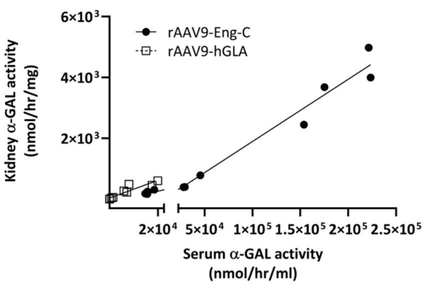

C

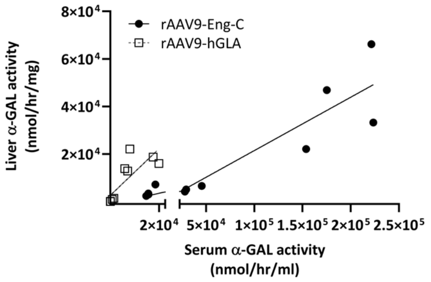

D

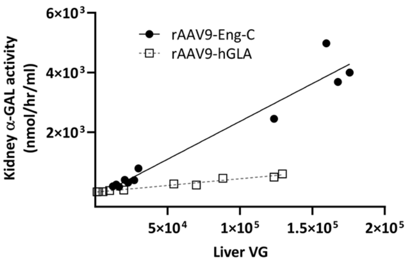

E

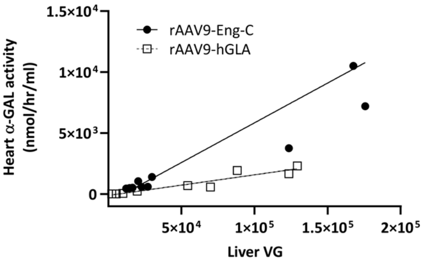

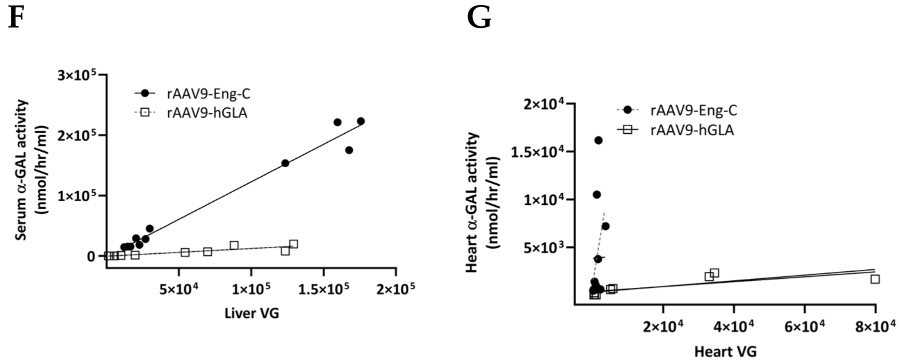

Figure S4. Correlation of serum α-GAL activity and liver vector genome (VG) copy number with tissue and serum α-GAL activity. (A, B, C) Correlation of serum α-GAL activity with α-GAL activity in the heart, kidney and serum. (D, E, F) Correlation of liver vector genome copy number with serum, heart, and kidney α-GAL activity. (G) Correlation of heart vector genome copy number and heart α-GAL activity. GraphPad Prism 10.2.1 Pearson function was used for correlation analysis and simple linear regression function was used to estimate the regression slope (Tables S2, S3).

	**Serum vs Heart α-GAL activity (nmol/hr/ml)**		**Serum vs Kidney α-GAL activity (nmol/hr/ml)**		**Serum vs Liver α-GAL activity (nmol/hr/ml)**	
	**rAAV9-Eng-C**	**rAAV9-hGLA**	**rAAV9-Eng-C**	**rAAV9-hGLA**	**rAAV9-Eng-C**	**rAAV9-hGLA**
**Correlation Pearson r**	0.89	0.99	0.99	0.93	0.91	0.81
95% confidence interval	0.61 to 0.97	0.97 to 1.0	0.95 to 1.0	0.72 to 0.98	0.69 to 0.98	0.37 to 0.95
R squared	0.78	0.99	0.97	0.86	0.83	0.66
P (two-tailed)	0.0003	<0.0001	<0.0001	<0.0001	<0.0001	0.00
**Regression Model**						
Slope	0.05	0.11	0.02	0.03	0.22	0.98
Goodness of Fit						
R squared	0.78	0.99	0.78	0.99	0.83	0.66
Slope difference	Not significant p=0.5		Not significant p=0.44		Significant, p=0.048	

Table S2. Serum and tissue α-GAL activity correlation. GraphPad Prism 10.2.1 Pearson function was used for correlation analysis and simple linear regression function was used to estimate the regression slope.

	Liver VG copy number vs. Serum α-GAL activity (nmol/hr/ml)		Liver VG copy number vs. Heart α-GAL activity (nmol/hr/ml)	
	**rAAV9-Eng-C**	**rAAV9-hGLA**	**rAAV9-Eng-C**	**rAAV9-hGLA**
**Correlation Pearson r**	0.99	0.87	0.87	0.94
95% confidence interval	0.95 to 1.0	0.54 to 0.97	0.56 to 0.97	0.74 to 0.99
R squared	0.98	0.76	0.75	0.89
P (two-tailed)	<0.0001	0.001	0.0005	0.0001
**Regression Model**				
Slope	1.24	0.13	0.07	0.02
Goodness of Fit				
R squared	0.98	0.76	0.75	0.89
Slope difference	Significant p<0.0001		Significant p=0.014	
	**Liver VG copy number vs. Kidney α-GAL activity (nmol/hr/ml)**		**Heart VG copy number vs. Heart α-GAL activity (nmol/hr/ml)**	
	**rAAV9-Eng-C**	**rAAV9-hGLA**	**rAAV9-Eng-C**	**rAAV9-hGLA**
**Correlation Pearson r**	0.97	0.98	0.44	0.77
95% confidence interval	0.8952 to 0.9931	0.9251 to 0.9960	-0.21 to 0.82	0.22 to 0.95
R squared	0.95	0.97	0.20	0.59
P (two-tailed)	<0.0001	<0.0001	0.17	0.02
**Regression Model**				
Slope	0.03	0.005	2.23	0.026
Goodness of Fit				
R squared	0.95	0.97	0.20	0.59
Slope difference	Significant p<0.0001		Borderline Significant p=0.07	

Table S3. VG and α-GAL activity correlation. GraphPad Prism 10.2.1 Pearson function was used for correlation analysis and simple linear regression function was used to estimate the regression slope.

Treatment	Mouse #	Lyso-Gb3 level (ng/mL serum or ng/mg tissue protein)			
		Serum	Kidney	Heart	Liver
rAAV9-Eng-C 2.5 x 10^{11} vg/kg	D1	0.72	0.08	0.26	0.14
	D2	0.69	0.05	0.07	0.12
	D3	0.52	0.05	0.08	0.12
	D4	0.58	0.04	0.11	0.12
	D5	0.56	0.05	0.06	0.12
AAV9-hGLA 2.5 x 10^{11} vg/kg	E1	2.52	0.45	5.25	0.66
	E2	1.97	0.17	5.43	0.48
	E3	2.42	0.2	3.6	0.53
	E4	2.48	0.39	6.56	0.71
	E5	1.64	0.3	3.16	0.45
rAAV9-Eng-C 5 x 10^{10} vg/kg	J1	0.41	0.09	0.5	0.11
	J2	0.46	0.08	0.15	0.1
	J3	3.02	0.11	0.52	1.14
	J4	0.69	0.12	0.62	0.17
	J5	0.81	0.14	1.49	0.21
	J6	0.66	0.07	0.32	0.16
AAV9-hGLA 5 x 10^{10} vg/kg	K1	4.44	1.7	10.37	1.88
	K2	23.2	3.68	9.71	7.06
	K3	3.54	0.77	12.4	0.94
	K4	61	6.43	12.4	17.65
	K5	59	5.47	16.35	14.63
	K6	23.6	4.13	12.25	8.8
Null vector control 2.5 x 10^{11} vg/kg	F1	301	10.41	18.27	67.82
	F2	237	9.35	24.18	74.78
	F3	217	7.85	15.33	62.71
	F4	270	8.04	15.19	68.99
	F5	284	6.46	24.47	57.06
WT control	L1	2.88	0.04	BQL	0.09
	L2	3.34	0.04	BQL	0.09
	L3	2.52	0.04	BQL	0.07
	L4	2.68	0.04	BQL	0.07
	L5	3.08	0.04	BQL	0.09
	L6	2.84	0.03	BQL	0.07

Table S4. Serum and tissue lyso-Gb3 level. BQL is below quantification limit.

		**Serum α-GAL Conc (ng/mL serum)**						**Tissue α-GAL Conc (ng/mg pr)**		
**Treatment**	**Mouse #**	**2-weeks post dose**	**4-weeks post dose**	**8-weeks post dose**	**12-weeks post dose**	**16-weeks post dose**	**18-weeks post dose**	**Kidney**	**Heart**	**Liver**
**rAAV9-Eng-C 2.5 x 10^11 vg/kg**	9	5203.9	7489.3	10116.3	11401.5	7331.1	7985.7	283.1		3094.8
	10	724.2	326.2	79.7	123.5	17.8	15.5	102.6		13.4
	17	27469.6	6871.5	26153.8	32915.0	37532.3	40232.8	953.0	8276.4	67603.1
	18	41649.7	48164.3	52180.1	69331.6	64389.3	57263.3	832.7	11366.7	55916.6
	19	108305.1	120910.0	94611.5	129342.0	66242.0	58778.5	1562.4		69387.6
	39	68.5	12.6	38.4	48.0	49.2	85.0	30.8		723.2
	40	795.1	452.9	528.9	798.1	983.0	837.8	50.8		2008.9
	41	94565.3	114803.6	82428.4	108588.4	88230.5	80767.7	2117.2	18950.5	46970.3
	42	71182.3	92003.4	76137.1	90980.6	69201.4	75094.7	1915.2	19306.0	53535.7
	43	67295.2	92293.2	71477.3	90581.5	62991.9	89048.0	2541.1	12180.6	70656.5
	100	9669.1	958.6	1607.6	BQL	7475.8	3662.8	149.9		15403.5
**rAAV9-Eng-C 5 x 10^10 vg/kg**	29	2761.0	3886.3	2589.1	3085.2	1665.3	1426.7	85.6		373.2
	31	1234.2	1283.5	1149.4	1284.9	176.0	545.8	13.7		211.5
	32	4147.7	5700.2	3324.5	4229.3	3954.6	3903.3	63.8		2032.8
	52	5151.9	4301.8	3989.4	3949.5	3881.7	3981.8	112.6		4580.5
	54	60.3	BQL	BQL	BQL	BQL	BQL	0.7	2.9	BQL
	55	3416.7	1639.7	65.5	BQL	BQL	2.6	0.9	2.4	BQL
	102	1435.2	769.2	363.2	676.2	991.9	1249.2	17.4	47.1	734.5
**Null vector control 2.5 x 10^11 vg/kg**	1						BQL	BQL		BQL
	2							BQL		BQL
	3						BQL	BQL	BQL	BQL
	46						BQL	BQL	BQL	BQL
	47						BQL	BQL	BQL	BQL
	65						BQL	BQL		BQL
	66						BQL	BQL	BQL	BQL
	97						BQL	BQL		BQL
	98						BQL	BQL		BQL
**WT control**	83						BQL	BQL		BQL
	84						BQL	BQL		BQL
	85						BQL	BQL		BQL
	86						BQL	BQL	BQL	BQL
	87						BQL	BQL	BQL	BQL
	88						BQL	BQL	BQL	BQL
	89						BQL	BQL	BQL	BQL
	90						BQL	BQL		BQL
	91						BQL	BQL		BQL

92							BQL		BQL
93						BQL	BQL	BQL	BQL
94						BQL	BQL	BQL	BQL

Table S5. Serum and tissue α-GAL protein. BQL is below quantification limit.

**Treatment**	**Mouse #**	**Serum (ng/ml)**	**Kidney (ng/mg protein)**	**Heart (ng/mg protein)**	**Liver (ng/mg protein)**
**rAAV9-Eng-C 2.5 x 10^{11} vg/kg**	9	4.08	0.07		0.08
	10	135.80	4.71		3.78
	17	2.16	0.03	BQL	0.03
	18	1.62	0.03	BQL	0.02
	19	2.66	0.13		0.03
	39	84.60	2.68		1.69
	40	11.22	0.29		0.20
	41	1.92	0.03	BQL	0.03
	42	3.72	0.05	0.02	0.06
	43	3.08	0.05	BQL	0.04
	100	4.10	0.07		0.09
**rAAV9-Eng-C 5 x 10^{10} vg/kg**	29	3.81	0.10		0.12
	31	11.85	0.19		0.28
	32	2.68	0.05		0.07
	52	1.83	0.06		0.05
	54	333.50	10.71	7.09	10.60
	55	225.00	5.30	6.13	6.97
	102	8.85	0.16	1.89	0.13
**Null vector control 2.5 x 10^{11} vg/kg**	1	860.00	41.69		127.74
	2	645.00	47.63		83.84
	3	452.50	30.41	19.20	85.39
	46	655.00	43.53	29.74	155.43
	47	780.00	41.39	21.82	144.38
	65	805.00	23.31		92.15
	66	745.00	22.21	15.17	89.33
	97	855.00	35.11		112.89
	98	630.00	9.20		27.45
**WT control**	83	1.99	0.08		0.03
	84	2.00	0.22		0.03
	85	1.89	0.21		0.04
	86	BQL	0.03		

	87	2.26	0.04	BQL	0.03
	88	2.02	0.06	BQL	0.04
	89	2.20	0.06	BQL	0.03
	90	2.32	0.04		0.05
	91	1.85	0.16		0.04
	92		0.05		0.03
	93	2.04	0.05	BQL	0.03
	94	1.82	0.09	BQL	0.04
**Pre-treatment**	23	472.50	4.37		37.45
	24	545.00	6.27		43.38
	25	499.00	4.49		40.33
	26	384.50	6.60	4.90	28.98
	27	487.50	5.08	5.41	39.84
	28	510.00	3.48	8.45	33.06

Table S6. Serum and tissue lyso-Gb3. BQL is below quantification limit.

	**Kidney α-GAL activity (nmol/hr/ml) vs Kidney Gb3 (ug/mg protein)**	**Serum α-GAL activity (nmol/hr/ml) vs Kidney Gb3 (ug/mg protein)**
**Correlation Pearson r**	-0.450	-0.554
95% confidence interval	-0.8414 to 0.2502	-0.8660 to 0.0688
R squared	0.203	0.309
P (two-tailed)	0.192	0.071
**Correlation Spearman r**	-0.685	-0.936
95% confidence interval		-0.9843 to -0.7592
P (two-tailed)	0.035	<0.0001

Table S7. Kidney and serum α-GAL activity and kidney Gb3 correlation. Correlation was performed in GraphPad Prism 10.2.1. Data for animals treated with both doses of rAAV9-Enz-C were included, n=10. Samples where Gb3 was reduced below normal level were excluded. Serum α-GAL activity at study midpoint (8 weeks) was used for correlation.

	**Heart α-GAL activity (nmol/hr/ml) vs Heart Gb3 (ug/mg protein)**	**Serum α-GAL activity (nmol/hr/ml) vs Heart Gb3 (ug/mg protein)**

**Correlation Pearson r**	-0.795	-0.737
95% confidence interval	-0.9611 to -0.2044	-0.9488 to -0.06624
R squared	0.631	0.543
P (two-tailed)	0.019	0.037
**Correlation Spearman r**	-0.881	-0.786
95% confidence interval		
P (two-tailed)	0.007	0.028

Table S8. Heart and serum α-GAL activity and heart Gb3 correlation. Correlation was performed in GraphPad Prism 10.2.1. Data for all animals treated with both doses of rAAV9-Eng-C were included, n=8. Serum α-GAL activity at study midpoint (8 weeks) was used for correlation.

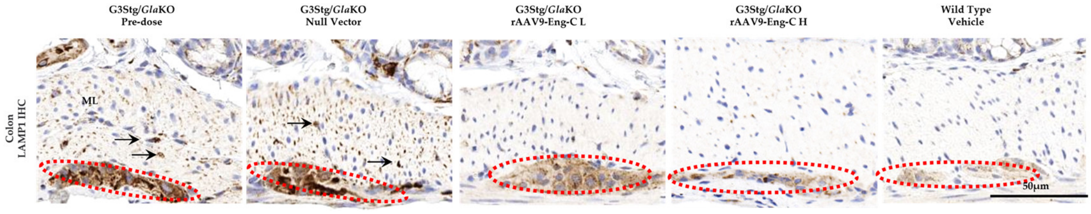

Figure S5. Immunostaining with anti-LAMP1 antibodies. Increased LAMP1 immunostaining intensity (brown) was observed in the myenteric plexus ganglia (MPG) and smooth muscles in the G3Stg/*Gla*KO mice colon at baseline. In mice treated with null vector accumulation persisted through the end of the study. Treatment with rAAV9-Eng-C reduced LAMP1 intensity from baseline in the low-dose group and normalized to the wild-type level in the high-dose group.

	**Serum α–GAL activity (nmol/hr/ml) vs MPZ LAMP1 positivity**	**Serum α–GAL activity (nmol/hr/ml) vs DRG LAMP1 positivity**
**Correlation Pearson r**	-0.700	-0.531
95% confidence interval	-0.9407 to 0.008557	-0.7995 to -0.08490
R squared	0.491	0.282
P (two-tailed)	0.053	0.023
**Correlation Spearman r**	-0.738	-0.761
95% confidence interval		-0.9085 to -0.4435
P (two-tailed)	0.046	0.0002

Table S9. Serum α-GAL activity and MPZ and DRG LAMP1 positivity correlation. Correlation was performed in GraphPad Prism 10.2.1. Data for animals treated with both doses of rAAV9-Eng-C were included in analysis, n=8. Serum α-GAL activity at study midpoint (8 weeks) was used for correlation.

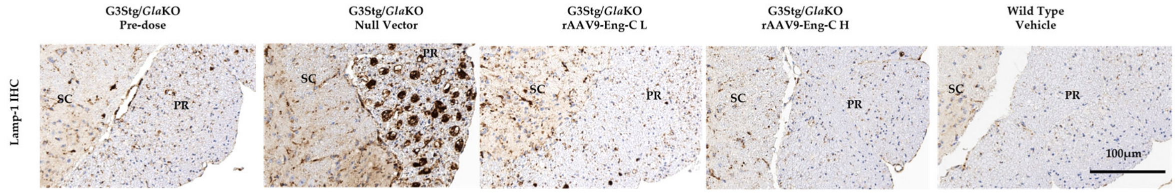

Figure S6. Immunostaining with anti-LAMP1 antibodies. Small increase in LAMP1 positivity (brown) in the nerve fibers of the posterior/dorsal root of the spinal cord was observed in G3Stg/*Gla*KO mice at baseline, in the pathology continued to progress in the null vector control group. Highest LAMP1 positivity was observed in the clusters of unmyelinated axons. Slight increase in LAMP1 positivity was also observed in the dorsal horn of the spinal cord. Treatment with low dose of rAAV9-Eng-C prevented further progression and high dose reversed pre-existing pathology. SC - Spinal cord; PR Posterior/Dorsal Root

Fisher's LSD	Mean Diff. (seconds)	95.00% CI of diff.	Summary	P Value
**Baseline**				
**WT control vs. rAAV9-Eng-C 2.5 x 10^{11} vg/kg**	-1.191	-4.773 to 2.392	ns	0.4965
**WT control vs. rAAV9-Eng-C 5 x 10^{10} vg/kg**	-3.81	-7.942 to 0.3219	ns	0.0685
**WT control vs. Null vector**	-5.556	-10.14 to -0.9762	*	0.0203
**2 weeks**				
**WT control vs. rAAV9-Eng-C 2.5 x 10^{11} vg/kg**	-3.364	-6.402 to -0.3250	*	0.0317
**WT control vs. rAAV9-Eng-C 5 x 10^{10} vg/kg**	-1.729	-6.578 to 3.121	ns	0.4482
**WT control vs. Null vector**	-4.903	-9.499 to -0.3066	*	0.0379
**4 weeks**				
**WT control vs. rAAV9-Eng-C 2.5 x 10^{11} vg/kg**	-1.042	-5.595 to 3.512	ns	0.6346
**WT control vs. rAAV9-Eng-C 5 x 10^{10} vg/kg**	-1.656	-6.705 to 3.393	ns	0.4801

**WT control vs. Null vector**	-8.083	-14.86 to -1.308	*	0.023
**8 weeks**				
**WT control vs. rAAV9-Eng-C 2.5 x 10^{11} vg/kg**	-0.9682	-4.795 to 2.858	ns	0.5946
**WT control vs. rAAV9-Eng-C 5 x 10^{10} vg/kg**	-2.393	-7.567 to 2.782	ns	0.3124
**WT control vs. Null vector**	-12.65	-17.52 to -7.782	***	0.0001
**12 weeks**				
**WT control vs. rAAV9-Eng-C 2.5 x 10^{11} vg/kg**	-0.4841	-3.705 to 2.737	ns	0.7525
**WT control vs. rAAV9-Eng-C 5 x 10^{10} vg/kg**	-2.975	-10.23 to 4.284	ns	0.3611
**WT control vs. Null vector**	-28.6	-40.17 to -17.03	***	0.0003
**16 weeks**				
**WT control vs. rAAV9-Eng-C 2.5 x 10^{11} vg/kg**	-4.25	-15.47 to 6.970	ns	0.4202
**WT control vs. rAAV9-Eng-C 5 x 10^{10} vg/kg**	0.35	-5.076 to 5.776	ns	0.8842
**WT control vs. Null vector**	-35.01	-50.39 to -19.63	**	0.0013

Table S10. Hot plate latency test results. G3Stg/*Gla*KO mice were treated with rAAV9-Eng-C or null vector control. Latency for each G3Stg/*Gla*KO group was compared to wild-type control. One-way ANOVA followed by Fishers Least Significant Difference (LSD) test was performed for each time point using in GraphPad Prism 10.2.1, ns > 0.05, * $p < 0.05$, ** $p < 0.01$, *** $p < 0.001$.

	**Serum α-GAL activity (nmol/hr/ml) vs serum lyso-Gb3 (ng/ml)**
**Correlation Pearson r**	-0.522
95% confidence interval	-0.8544 to 0.1131
R squared	0.273
P (two-tailed)	0.099

**Correlation Spearman r**	-0.900
95% confidence interval	-0.9750 to -0.6404
P (two-tailed)	0.0004

Table S11. Serum α-GAL activity and serum lyso-Gb3 correlation. Correlation was performed in GraphPad Prism 10.2.1. Data for all animals treated with both doses of rAAV9-Eng-C were included, n=13. Serum α-GAL activity at study midpoint (8 weeks) was used for correlation.

Animal ID	Cmax (at 2 weeks) Serum α-GAL ng/ml	Baseline (Day 1) Total anti-AAV9 titer	Baseline (Day 1) Neutralizing anti-AAV9 titer	Anti-Eng-C α-GAL titer	Day detected
	**6.25 x 10^{12} vg/kg Group**				
2001	23800	Negative	5	120	21
2501	3320	Negative	20	120	28
2502	2320	400	20	3240	21
	**3 x 10^{13} vg/kg Group**				
3001	3740	400	10	9720	28
3002	BLQ	6400	320	ND	ND
3003	38100	Negative	20	120	21
3501	BLQ	51200	640	40	21
3502	18500	Negative	20	120	21
3503	12600	50	20	29160	28
3504	245000	Negative	10	360	42

Table S12. α-GAL concentration at 2 weeks, anti-AAV9 and anti-Eng-C variant in NHP serum at indicated day after treatment administration.

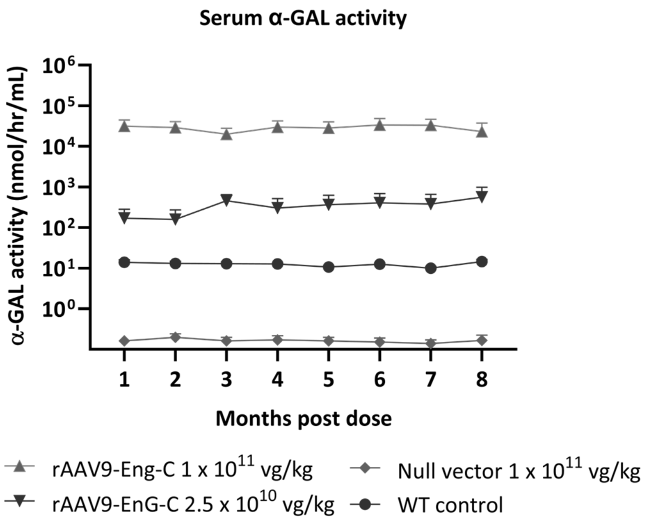

Figure S7. Serum α-GAL activity overtime. 8 to 12 weeks old *Gla*KO male mice were injected with AAV test article at indicated doses or null vector control. Wild-type mice (WT) injected with buffer were used as normal control. Serum samples were collected at 1, 2, 3, 4, 5, 6, 7 and 8 months after dosing. Activity was measured using 4-MU-α-gal fluorescent substrate. N = 3 to 10 per group/per timepoint

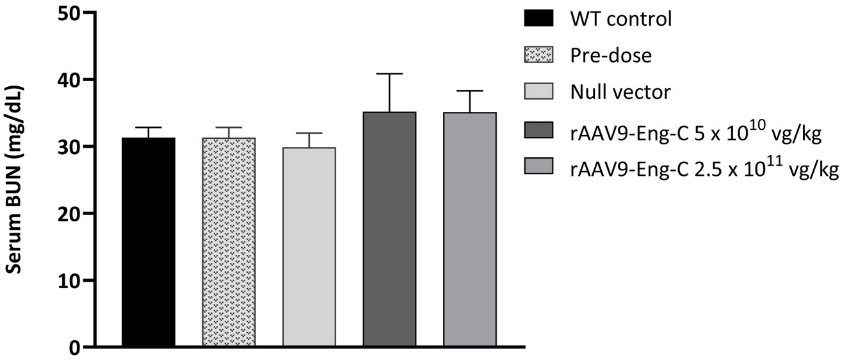

Figure S8. Blood Urea Nitrogen (BUN) in the pre-dose and terminal serum samples. N = 7 to 11 per group.

Animal ID	Treatments	Pre-Dose	Day 3	Day 7	Day 14	Day 21	Day 28
1001	Formulation buffer	>1:20	>1:40	>1:40	>1:40	>1:20	>1:40
1501		>1:40	>1:80	>1:80	>1:80	>1:80	>1:80
2001	6.25 x 10^{12} vg/kg (1-month)	>1:5	>1:40	>1:80	>1:160	>1:320	>1:320
2501		>1:20	>1:40	>1:320	>1:320	>1:160	>1:160
2502		>1:20	>1:40	>1:2560	>1:5120	>1:640	>1:640
3001	3 x 10^{13} vg/kg (1-month)	>1:10	>1:10	>1:640	>1:5120	>1:2560	>1:2560
3501		>1:640	>1:640	>1:2560	>1:2560	>1:2560	>1:2560
3502		>1:20	>1:40	>1:640	>1:640	>1:320	>1:1280
3002		>1:320	>1:320	>1:1280	>1:2560	>1:2560	>1:5120
3003		>1:20	>1:40	>1:320	>1:320	>1:640	>1:1280

3503	3 x 10^{13} vg/kg (3-month)	>1:20	>1:40	>1:640	>1:640	>1:5120	>1:5120
3504		>1:10	>1:20	>1:640	>1:640	>1:640	>1:1280

Table S13. Anti-rAAV9 neutralizing antibodies titers in the NHP study of each subject throughout the study.
